# *Nocardia fodinahabitans* sp. nov. isolated from underground hard coal mine waters shows biotechnological potential for degradation of aromatic hydrocarbons

**DOI:** 10.1007/s00438-026-02456-6

**Published:** 2026-06-16

**Authors:** Julia Marciniak, Trine Sørensen, Agnieszka Nowak, Magdalena Noszczyńska, Jakub Smoliński, Katarzyna Machnik, Karolina Solska, Mateusz Pala, Frederik T. Hansen, Teis Søndergaard, Mariola Paściak, Piotr Siupka

**Affiliations:** 1 Institute of Biology, Biotechnology and Environmental Protection, Faculty of Natural Sciences, University of Silesia in Katowice, Katowice, Poland; 2 Faculty of Engineering and Science, Department of Chemistry and Bioscience, Aalborg University, Aalborg, Denmark; 3 Hirszfeld Institute of Immunology and Experimental Therapy, Polish Academy of Sciences, Wroclaw, Poland; 4 Present Address: Department of Mathematical Sciences, Faculty of Engineering and Science, Aalborg University, Aalborg, Denmark; 5 Idun Biologics ApS, Aalborg, Denmark

**Keywords:** *Nocardia*, *Actinomycetota* of coal-related environments, Microorganisms from coal mines, Biodegradation of aromatic compounds, Chemotaxonomy, Whole genome sequencing, Biosynthetic gene clusters

## Abstract

The aim of the study was to describe and evaluate the biotechnological potential of a novel species of *Nocardia* isolated from underground coal mine water. *Nocardia* strains represent important human pathogens, but are also common in soil and water environments. Recently, they have gained attention for their biosynthetic potential and vast metabolic abilities to degrade aromatic compounds, including xenobiotics. In this work, we established the taxonomic status of strain MW-W600-9^T^. Phylogenetic analysis based on 16 S rRNA gene and whole genome sequencing revealed close relatedness to *Nocardia rhizosphaerihabitans* and *Nocardia asteroides*. This is further supported by phenotyping tests, chemotaxonomic analyses and MALDI-ToF profiling. We therefore propose that strain MW-W600-9^T^ (= DSM 120649^T^ =PCM 3565^T^) constitutes a new species, *Nocardia fodinahabitans* sp. nov. The strain has 31 putative biosynthetic gene clusters, 12 showing low or no similarity to the clusters of known compounds, indicating its potential to produce novel bioactive compounds. The strain MW-W600-9^T^ shows degradation potential of aromatic xenobiotics, tested for haloorganic compounds and bisphenols. The concentration of 4-chlorophenol and bisphenol A was decreasing significantly during the strain culture, and the results suggest co-metabolic removal of iohexol. This work shows the biodegradation ability of aromatic xenobiotic compounds by a new species of *Nocardia*, highlighting the potential use of the strain in bioremediation applications. It also shows for the first time the degradation of bisphenol by a member of the *Nocardia* genus, along with potential co-metabolic degradation of environmentally persistent haloorganic compounds.

**Supplementary Information:**

The online version contains supplementary material available at 10.1007/s00438-026-02456-6.

## Introduction

The scope of this study is to describe the new species of *Nocardia* isolated from underground coal mine water. The description is based on comparison of the isolated strain and related type strains at the molecular, phenotypic and metabolic levels. Furthermore, the study explores strain’s biotechnological potential for the degradation of aromatic organic xenobiotics, highlighting its possible use in bioremediation of toxic and persistent contaminations of environment.

*Nocardia* is one of the genera in the phylum *Actinomycetota* (Oren and Garrity 2021) and a type genus of family *Nocardiaceae* (Zhi et al. 2009; Nouioui et al. 2018). Members of that genus are aerobic, Gram-stain-positive bacteria with complex, branching morphology forming pseudomycelium. They have large genomes of approx. 7 Mbp and G + C content in the range of 63–72 mol % (Barka et al. 2016). Chemotaxonomic and genome analyses enabled the distinction of *Nocardia* from other morphologically similar or mycolic acid-containing *Actinomycetota* (Conville et al. 2018; Tamura et al. 2018; Nouioui et al. 2022). They are characterized by *meso*-diaminopimelic acid (A2pm) as a diaminoacid of peptidoglycan, whole-organism sugars are arabinose and galactose, while straight-chain, saturated, unsaturated and 10-methyl components form their fatty acids. Major polar lipids are diphosphatidylglycerol, phosphatidylethanolamine, phosphatidylinositol and phosphatidylinositol mannosides. They have 46–64 carbon atoms mycolic acids with up to four double bonds. Menaquinones in most *Nocardia* are hexahydrogenated with eight isoprene units, in which the end two units are cyclized. The predominant respiratory quinone is MK-8(H4-ω-cyclo) (Goodfellow and Maldonado 2012; Tamura et al. 2018).

Most known species of *Nocardia* are associated with severe infections, predominantly of the respiratory tract, and are etiologically linked to nocardiosis, a condition that affects the respiratory tract, as well as the brain or skin. There are also disseminated forms of nocardiosis (Brown-Elliott et al. 2006; Mehta and Shamoo 2020). Among the most frequent are *Nocardia farcinica* and *Nocardia cyriacigeorgica* (Conville et al. 2018). Close to half of over 100 species validly named are recognized as pathogens or opportunistic pathogens (Traxler et al. 2022). Many of the *Nocardia* strains have also been isolated from natural habitats, including diverse soils and waters (Golinska et al. 2013; Wang et al. 2015; Bai et al. 2016; Sharma et al. 2016; Ding et al. 2018; Wright et al. 2023). They were also found in microbiomes of various plants (Tanvir et al. 2016; Ghodhbane-Gtari et al. 2019; Nouioui et al. 2022) as well as animals (Zhang et al. 2008; Thawai et al. 2017; Benndorf et al. 2020). Genome analysis of some of such species, like *Nocardia alni*, suggests their beneficial interaction with higher organisms (Nouioui et al. 2022), whereas for *N. cyriacigeorgica*, it has shown evidence for adaptation from a saprophytic to a pathogenic lifestyle (Zoropogui et al. 2013). Altogether, this shows that although they are mostly terrestrial bacteria, they have evolved a plethora of adaptations for interacting with higher organisms. Moreover, comparative genomics has shown that on average genomes of pathogenic species are slightly smaller than those of other strains and contain fewer biosynthetic gene clusters (BGCs) (Nouioui et al. 2022; Eripogu et al. 2025).

Biosynthetic potential for the production of novel secondary metabolites (SMs) is another characteristic feature of *Nocardia* as well as other taxa in the *Actinomycetota* phylum (Barka et al. 2016; Engelbrecht et al. 2021). Progress in analysis and comparative genomics in recent years revealed a high diversity of BGCs within the genus, comparable to that of the more extensively studied in that aspect, the *Streptomyces* genus (Männle et al. 2020; Eripogu et al. 2025). Furthermore, many of the discovered clusters are unique, making *Nocardia* a vastly unexplored source of metabolites. As studying the biosynthetic potential of *Nocardia* strains is a relatively recent approach, there are still many gaps in knowledge, as well as a bias toward pathogenic strains, as most isolates come from humans or animals, while environmental and saprophytic strains are underrepresented (Eripogu et al. 2025). The genus is already a source of important secondary metabolites like brasilicardin A (Shigemori et al. 1998), nocardicins (Kelly and Townsend 2005), amicoumacin B (Sun et al. 2009), or tubelactomicin A (Igarashi et al. 2000), just to name a few.

Another important feature for which members of *Nocardia* are known is their ability to utilize a plethora of complex and aromatic organic compounds as a source of carbon and energy (Luo et al. 2014; Barka et al. 2016; Shi et al. 2022). This was connected with strains’ adaptations to various environments, from which many have limited sources of easily accessible nutrients but are rich in complex organic compounds (Khomenkov et al. 2008; Nitika et al. 2019; Shi et al. 2022). Metabolic robustness, even among potentially pathogenic strains, has been utilized for their ability to degrade xenobiotics. Azadi and Shojaei (Azadi and Shojaei 2020) have shown potential for degradation of poly-aromatic hydrocarbons (PAHs) by several strains belonging to *N. farcinica*,* Nocardia kroppenstedtii* and *Nocardia fluminea*. The *N. fluminea* AN22 strain exhibited the highest degradation rate and was able to degrade up to 90% of PAHs supplemented to the culture media. Another strain, *Nocardia* sp. Y48 was able to perform almost complete mineralization of crude oil (Yang et al. 2019). *Nocardia nova* GTC 86116S^T^, isolated from active sludge, has shown potential to degrade benzene, toluene, ethylbenzene and xylene. The strain degraded 1500 mg/L of xylene during 96 h of incubation (Hocinat et al. 2019).

In the present study, we have isolated *Nocardia* strain MW-W600-9^T^ from the collective shaft coal mine water, established its taxonomical status as well as explored the strain’s biotechnological potential. The polyphasic approach, based on whole genome sequencing and chemotaxonomic analyses, showed it is a new species; the name proposed for the organisms is *Nocardia fodinahabitans* sp. nov., with strain MW-W600-9^T^ as the type strain. Moreover, we have analyzed the strain’s biosynthetic potential, tested its biological activity against bacteria and fungi, as well as assessed its ability to remove aromatic xenobiotics (bisphenols A and S, 4-chlorophenol and iohexol) from the culture medium.

## Materials and methods

### Isolation and maintenance of the strain, microscopy

Strain MW-W600-9^T^ was isolated from underground, collective shaft water (depth of 665–850 below ground level (bgl) sampled at a depth of 665 m bgl, as in (Siupka et al. 2021). Water was sampled in the Upper Silesian region in Poland (50°10’48” N, 19°5’47” E), taking advantage of the ongoing hard-coal mining activity. Potato-dextrose agar (PDA) plates were inoculated with 200 µL of the sample and incubated at 26 °C in the dark. Plates were investigated daily for microbial growth; any colonies that appeared were streak-inoculated on new PDA plates and kept in the same conditions in order to isolate pure cultures. For preservation, strain MW-W600-9^T^ was scraped off from the plate into potato-dextrose broth (PDB) supplemented with glycerol to a concentration of 30% and stored at −80 °C. Cells were stained using Gram’s method and observed under PrimoStar microscope (Carl Zeiss, Jena, Germany) with Primo Plan-ACHROMAT 100x NA 1.25 objective (Carl Zeiss, Jena, Germany) under immersion. The picture was taken using AxioCam ERc 5 s camera (Carl Zeiss, Jena, Poland).

### Microbial strains

Strains used for comparative chemotaxonomic study, *Nocardia asteroides* DSM 43757^T^ and *Nocardia rhizosphaerihabitants* DSM 101726^T^ were obtained from the German Collection of Microorganisms and Cell Cultures (DSMZ, Germany). Both strains were maintained on International Streptomyces Project medium 2 (ISP2) plates.

Strains used for antimicrobial tests included bacteria *Escherichia coli* ATCC 8739™, *Pseudomonas aeruginosa* ATCC 27853™, *Yersinia enterocolitica* (provided by Dr. Katarzyna Kasperkiewicz from the University of Silesia in Katowice), *Bacillus subtilis* (from the University of Silesia in Katowice collection), and *Staphylococcus aureus* subs. *aureus* ATCC 65389™, yeast *Candida albicans* ATCC 10231™ and mycelial fungus *Fusarium culmorum* DSM 62188 (from DSMZ, Germany). Bacteria and yeast strains were maintained on nutrient agar (NA) plates, while *F. culmorum* DSM 62188 was maintained on PDA plates. Reference strains were obtained from American Tissue and Cell Culture Collection (ATCC, USA) unless indicated otherwise.

### Growth conditions and metabolic properties

Growth of the strain MW-W600-9^T^ on different microbiological media was investigated by streak inoculation of the strain on agar plates, followed by incubation at 25 °C in the dark. Plates were investigated after 7 days, and growth was assessed visually. Growth on the Arginine-glycerol salt (AGS) (El-Nakeeb and Lechevalier 1963), International Streptomyces Project (ISP) medium 1 (ISP1), ISP2, ISP3, ISP4, ISP9, Actinomycete Isolation Agar (AIA), Sabouraud’s Agar, Löwenstein–Jensen’s Agar, Blood Agar, Columbia Blood Agar, BHI medium, medium 79 (Paściak et al. 2014), TGTS medium (Chudzik et al. 2022) and minimal salt medium supplemented with 2% of grounded coal (MSMA-C2) (Siupka et al. 2020) was investigated.

Growth of the strain MW-W600-9^T^ at different temperatures: 4 °C, 8 °C, 25 °C, 37 °C, 42 °C, 45 °C was examined by making streak inoculations of the single colony of strain on PDA and ISP2 plates using loop and incubation for 7 days in the dark. After that time, the plates were visually investigated for microbial growth. Plates with no growth were placed at 25 °C for the next 7 days of incubation to investigate strain viability. The anaerobic growth capability of the strain was assessed using the GasPack^®^ system (Becton, Dickinson and Company, Sparks, MD, USA) and the GENbox anaer generator (BioMérieux, France). Plates were incubated in the dark for 7 days.

Growth of the strain MW-W600-9^T^ at different pH and salinity levels was examined in potato dextrose broth (PDB) by inoculation of 6 ml of media with 50 µL of spores’ solution. The spores’ solution was prepared by harvesting the aerial mycelium from 2 PDA plates and suspending it in 4 ml of sterile water, followed by brief vortexing. Media aliquots have been adjusted for pH using 1 M HCl or 1 M NaOH; the growth was tested at the pH values: 4.0, 5.0, 6.0, 7.0, 8.0, 9.0, and 10.0. Salinity of the media was adjusted using 20% (w/v) NaCl solution. The growth was examined at NaCl concentrations: 0%, 0.5%, 1%, 1.5%, 2%, 4%, 6%, 8% and 10%. Tubes were incubated at 25 °C in the dark with rotatory shaking at 120 rpm. After 7 days of incubation, the growth of the strain MW-W600-9^T^ was visually assessed.

Metabolic and growth profiles of the strain MW-W600-9^T^, *N. asteroides* DSM 43757^T^, and *N. rhizosphaerihabitants* DSM 101276^T^ were compared using the GEN III MicroPlate™ assay (BiOLOG Inc., CA, USA) according to the manufacturer’s protocol. Briefly, biomass was scraped from a 7-day culture on PDA plates and suspended in IF-B inoculation fluid (BiOLOG Inc., CA, USA) until transmittance of 98%. Next 150 µL of inoculum was added per well of the microplate. For each strain, three independent plates were inoculated. Plates were incubated at 25 °C for 14 days, with absorbance at 495 nm reading done every 24 h on SPARK microplate reader (Tecan, Switzerland).

### Chemotaxonomy

The strains MW-W600-9^T^, *N. asteroides* DSM 43757^T^ and *N. rhizosphaerihabitans* DSM 101726^T^ were compared for chemotaxonomic properties that are of value in nocardial systematics (Conville et al. 2018; Benndorf et al. 2020; Nouioui et al. 2022). The strains were cultured in 100 mL of PDB and ISP2 medium for 7 days at 25 °C, 120 rpm in the dark. After that time, biomass was harvested by centrifugation, washed twice with phosphate-saline buffer (PBS), frozen in liquid nitrogen, lyophilized using Alpha 1-4LSCBasic lyophilizer (Christ, Germany) and used for subsequent analyses.

The fatty acid profile was analyzed by fatty acid methyl esters (FAMEs) extraction according to the method by Sasser (Sasser 2001). FAMEs profile analysis was conducted by gas chromatography on a 7829A device (Agilent Technologies, CA, USA) with a FID detector supplied with phenyl-methyl-silicate capillary column (diameter 0.22 mm, length 25 m, thickness 0.33 μm; Agilent Technologies, CA, USA). Injection volume was 2 µL, pressure was 10.7 psi (71.33 kPa). The carrier gas was hydrogen. During the analysis, the oven temperature is ramped from 170 °C to 260 °C at a rate of 5 °C/min. FAMEs were identified based on calibration standard No. 1300-C (MIDI, DE, USA). For each strain and media analyses were performed in triplicate.

The isomer of diaminopimelic acid (DAP) in whole-cell hydrolysates was determined following the method described by Staneck & Roberts (Staneck and Roberts 1974) and analyzed using thin-layer chromatography with a standard of 2,6-diaminopimelic acid. The presence of N-glycolyl groups in the peptidoglycan of the isolate was assessed according to the procedure outlined by Uchida et al. (Uchida et al. 1999).

Whole cell wall sugars were obtained using the method described by Lechevalier (Lechevalier and Lechevalier 1980). These sugars were then converted into alditol acetates following the procedure outlined by Sawardeker et al. (Sawardeker et al. 1965) and analyzed by gas chromatography-mass spectrometry (GC-MS). The sugar derivatives were examined using a Focus GC connected to an Ion Trap ITQ 700, employing a Zebron ZB-5HT column (30 m × 0.25 mm × 0.25 μm, Phenomenex). The temperature program used for the analysis ranged from 150 °C to 270 °C at a rate of 12 °C/min, with helium serving as the carrier gas.

Polar lipids were extracted from 50 mg of freeze-dried cells using the Bligh & Dyer method (Bligh and Dyer 1959). The extracted lipids were then separated by two-dimensional thin-layer chromatography (TLC) on 10 × 10 cm silica gel 60 HPTLC plates (Merck-Millipore, Germany). The first dimension utilized a solvent system of chloroform, methanol, and water in a ratio of 65:24:4 (v/v/v), the second dimension used a solvent mixture of chloroform, glacial acetic acid, methanol, and water in a ratio of 80:12:15:4 (v/v/v/v). The total lipid profile was visualized by spraying the plates with phosphomolybdic acid, followed by heating at 120 °C for 10 min. Phospholipids were specifically detected using Dittmer and Lester reagents (Dittmer and Lester 1964).

Mycolic acids were obtained through acid hydrolysis and by the alkaline method and analyzed using thin-layer chromatography (TLC) with nocardiomycolate standards (Embley and Wait 1994). Mycolic acid methyl esters from strain MW-W600-9^T^, as well as from *N. asteroides* DSM 43757^T^ and *N. rhizosphaerihabitants* DSM 101726^T^, were purified via column chromatography. These purified esters were then analyzed by MALDI-TOF mass spectrometry in reflectron positive-ion mode, using norharmane as the matrix (Machnik et al. 2024).

### MALDI-TOF mass spectrometry

Matrix-assisted laser desorption/ionization-time-of-flight (MALDI-ToF) mass spectra were generated from fresh cultures of the strains MW-W600-9^T^, *N. asteroides* DSM 43757^T^, and *N. rhizosphaerihabitans* DSM 101726^T^ obtained from PDA medium and next cultivated on nutrient agar (NA), sheep blood agar (BL), and yeast extract glucose agar (medium 79) (Paściak et al. 2014) for 3–4 days at 25 °C. For comparative purposes, nocardial strains were also grown under stationary conditions in 5 mL tubes for 3–4 days at 25 °C. Cells, grown on liquid 79 medium, were centrifuged (MiniSpin, Eppendorf, Hamburg, Germany) at 12,100 rcf for 2 min, the supernatant was removed, and the pellet was resuspended in 2 ml of PBS and centrifuged as above. Samples were prepared according to the ethanol–formic acid extraction, recommended by the manufacturer as described previously (Chudzik et al. 2024). MALDI-ToF MS analysis was conducted on the Ultraflex mass spectrometer (Bruker Daltonics, Germany) using Biotyper 3.1 software and a database containing 8469 entries. Spectra were recorded in the linear positive ion mode within a mass range of 2,000–20,000 Da. The mass spectra were externally calibrated using the *E. coli* DH5-alpha standard (Bruker Daltonics).

### DNA isolation, 16S rRNA gene and whole genome sequencing

Sequencing of the strain MW-W600-9^T^ 16S rRNA gene and the strain’s whole genome were performed.

Genomic DNA extraction was conducted from bacterial mass harvested from a culture in 50 mL PDB. After harvesting, biomass was lyophilized and DNA purification and quality assurance were conducted as described previously (Petersen et al. 2022). Extracted high molecular weight genomic DNA was used for polymerase chain reaction (PCR) in order to amplify 16S rRNA gene. The amplification, reaction verification, and product purification were performed as described before (Dzionek et al. 2025). Sanger sequencing of the amplicon in both forward and reverse direction using the same primers as for the PCR was carried out by Genomed S.A. (Warsaw, Poland). The poor-quality nucleotides were trimmed from both ends of the resulting sequences, and the partial 16S rRNA gene was reconstructed by overlapping the forward and reverse fragments of the gene. The trimming and reconstruction were done using Unipro Ugene software (version 51.1) (Okonechnikov et al. 2012). The reconstruction resulted in a 1260 bp sequence, which was deposited in the National Center for Biotechnology Information (NCBI) under accession number PX522239.

The whole genome sequence of the strain MW-W600-9^T^ was obtained using long-read nanopore sequencing, polished with short reads Illumina sequencing, similar to Siupka et al. (Siupka et al. 2021). For nanopore sequencing, the SQK-LSK109 kit (Nanopore Technologies, Oxford, UK) was used to perform barcoding of 400 ng of high molecular weight genomic DNA using the EXP-NBD104 barcodes. Then, it was loaded onto the R49 flow cell (Nanopore Technologies, Oxford, UK). Data acquisition was done using MinKNOW (Nanopore Technologies, Oxford, UK), and base calling and demultiplexing were done with Guppy v 4.2.2 (Nanopore Technologies, Oxford, UK). The trimming of reads was performed using Filtlong v 0.2.0 (https://github.com/rrwick/Filtlong; accessed on 9 March 2020) followed by genome assembling using Miniasm v 0.3 and minimap2 v 2.17 (Li 2016). The resulting assembly was polished using short-read data by Racon v 1.3.3 (Vaser et al. 2017) and Medaka v 1.0.1 (https://github.com/nanoporetech/medaka; accessed on 9 March 2020). The quality of the assembled genome was evaluated using the Comprehensive Genome Evaluation tool at the BV-BRC server (accessed on 10th March 2022)(Wattam et al. 2017).

For Illumina sequencing, the strain was spread-inoculated on PDA plates and cultivated for 10 days, as described above. After incubation, cells were scraped from the plate and sent to MicrobesNG (Birmingham, UK). The sequencing was performed using the Illumina MiSeq platform (Illumina, San Diego, CA, USA) with 2 × 250 bp pair-end reads. The results were processed via the standard MicrobesNG analysis pipeline (Birmingham, UK).

The assembly was deposited in the National Center for Biotechnology Information (NCBI) Genbank database at the accession number: JBRJJC000000000.

### Phylogeny

A partial 16S rRNA gene was used for phylogenetic analysis of the strain MW-W600-9^T^. The sequences were searched against the NCBI 16S rRNA/ITS database that includes only sequences from the type strains using the BLAST tool (Eric et al. 2014). Sequences from the top 100 hits were downloaded and used for alignment and phylogenetic tree calculation. Additionally, two sequences from *Mycobacterium marinum* ATCC 927^T^ and *Mycobacterium vulneris* DSM 45247^T^ were included as an outgroup. The sequence alignment was done using the MAFFT algorithm with default settings (Katoh et al. 2002) in Unipro Ugene software. The resulting alignment was used for phylogenetic tree calculation using IQ-TREE2 software (version 2.4.0) (Minh et al. 2020) with automatic substitution model selection (Kalyaanamoorthy et al. 2017). The model was validated using 1000 ultrafast bootstraps (Minh et al. 2013). The tree was visualized using the Interactive Tree of Life server (Letunic and Bork 2024).

Phylogenomic analysis of the strain MW-W600-9^T^ was done based on the whole genome sequence. The genome was subjected to comparison against the type strain database using digital DNA-DNA hybridization (dDDH) and Type Genome Server (TYGS) (Meier-Kolthoff and Göker 2019). Average nucleotide identity between the strain genome and related genomes was calculated using Tetra and ANIb tools at the JSpeciesWS server (Richter et al. 2016). The initial results from the servers were used to compile a database of closely related genomes, and another round of analysis on both servers was performed using unified input.

The phylogenetic tree was calculated based on the aligned core proteome of the strain and 259 high-quality genomes elucidated using the M1CR0B1AL1Z3R server (Avram et al. 2019). 257 high-quality genomes of *Nocardia* were used for core proteome prediction, and 2 *Mycobacterium* genomes were obtained from the BV-BRC database (accessed on 10th of July 2022) and used as an outgroup. The poorly aligned blocks of core proteome alignment were removed using the Gblock tool v 0.91b (Talavera and Castresana 2007). A maximum-likelihood tree was calculated using IQ-TREE2 software (Minh et al. 2020) using model finder (Kalyaanamoorthy et al. 2017) and validation by performing 1000 ultra-fast bootstraps. The tree was visualized using the interactive Tree of Life server (Letunic and Bork 2019).

### Annotation and putative biosynthetic gene clusters analysis

Gene prediction and functional annotations of the strain MW-W600-9^T^ genome were performed using eggNOG-mapper v 2.1.12 (Cantalapiedra et al. 2021) with DIAMOND tool (Buchfink et al. 2021) and BlastKoala (Kanehisa et al. 2016). Predicted and functionally annotated genes were mapped against Kyoto Encyclopedia of Genes and Genomes (KEGG) (Yi et al. 2020) using KEGG Mapper Reconstruct (Kanehisa et al. 2022). The annotations were manually searched for the genes connected with aromatic compounds degradation based on literature information.

Putative biosynthetic gene clusters of the strain MW-W600-9^T^ and 26 closely related strains were predicted using the antiSMASH tool v 6.1 (Blin et al. 2017) in relaxed mode. Results were manually investigated because a single region might contain more than one cluster. The “neighboring clusters” were not included in the final count; instead, only single clusters were included. A cluster was counted as a hybrid cluster only when antiSMASH classified it as such.

### Antimicrobial activity examination

Antimicrobial activity of the strain MW-W600-9^T^ against unicellular organisms, bacteria and yeast was performed using the disk diffusion assay. For that, 100 mL of PDB medium was loop inoculated with a 7-day-old culture of the strain on PDA and incubated for 14 days at 25 °C, 120 rpm in the dark. Next, the organic fraction was extracted twice using a 1:1 culture and extraction mixture containing ethyl acetate, dichloromethane, methanol (3:2:1, v/v/v) supplemented with 1% formic acid. After extraction, the solvent was evaporated on a rotatory evaporator, KNF S.C 920 (Heidolph, Germany) with final drying under nitrogen. Microorganisms for the test were cultured in 35 ml of nutrient broth until optical density at λ = 600 nm (OD600) reached 0.5. Next, 100 µL of each microorganism’s culture was lawn inoculated on Muller-Hinton agar (MHA) plates. The crude extract was dissolved in methanol, and 50 µL was transferred onto sterile 5 mm paper disks, left for a while to soak, and next the disks were placed on the inoculated plates. As controls, disks soaked with extract from abiotic PDB culture medium and methanol were used. Plates were incubated overnight at 25 °C. After incubation, pictures of the plates have been taken, and clear zones around the well were measured using Fiji software (Schindelin et al. 2012). The experiment has been performed in triplicate.

Antifungal activity against mycelial fungi has been tested against *F. culmorum* DSM 62188 as described previously (Siupka et al. 2021). Briefly, the strain MW-W600-9^T^ was line inoculated on MHA plates from 7 days old PDA plate, at a distance of 25 mm from the test plate center. Plates were incubated for 3 days at 25 °C in the dark. Next, an 8 mm puck with mycelium cut-out from a 10-day-old fungus culture on a PDA plate was placed at the center of the plates. Plates were further incubated at 25 °C in the dark. As a control, plates prepared in the same way, but without the strain MW-W600-9^T^ inoculation, were used. After 4 days of co-culture, pictures of the plates were taken, and the growth of the fungus toward the strain colony and in a parallel direction were measured using Fiji software.

### Aromatic compounds biotransformation

Potential of the strain MW-W600-9^T^ for the removal of aromatic compounds from liquid cultures was investigated for bisphenol A (BPA), bisphenol S (BPS), 4-chlorophenol (4CP) and iohexol (IOX). For bisphenols, the strain was cultivated for 7 days on Reager’s 2A (R2A) plates at 25 °C in the dark, followed by loop inoculation on liquid R2A supplemented with BPA or BPS at a concentration of 10 mg/L. The cultures were incubated at 25 °C, 120 rpm in the dark for 21 and 18 days for BPA- and BPS-supplemented cultures, respectively. As a control, abiotic cultures were prepared in the same way. Culture samples were collected every 3 days. Cells were removed by centrifugation (16,000 rcf, 15 min, 4 °C) and bisphenols were extracted from culture media using solid phase extraction (SPE) with Strate C18-E (55 µM, 70 Å, 100 mg/mL) columns (Phenomenex, CA, USA). Extraction was performed by activation of the sorbent with 4 ml of 70% EtOH, 4 ml of H_2_O, followed by the addition of 2 ml of sample and elution in 2 ml of 70% EtOH. Eluates were filtered through 0.22 μm regenerated cellulose syringe filters (Hahnemühle, Germany). Bisphenols concentration was analyzed using reverse-phase high-performance liquid chromatography (RP-HPLC) as described previously (Noszczyńska et al. 2023).

For the removal of 4CP and IOX, the strain was cultured in a similar way, but the media were supplemented with either 4CP (250 mg/L), IOX (50 mg/L) or 4CP (250 mg/L) + IOX (50 mg/L). As a control, abiotic cultures with 4CP (250 mg/L) or IOX (50 mg/L) have been prepared. As these compounds were expected to be more resistant to degradation, the culture was conducted for 31 days, and samples were collected on days 0, 17 and 31. For analysis of 4CP and IOX concentration, 1.5 mL of the culture was collected and centrifuged at 16,000 rcf, 4 °C for 15 min. Next, 1 mL of supernatant was transferred to new tubes and 10 µL of glacial acetic acid was added to prevent subsequent degradation of aromatic compounds. Concentration of both compounds was analyzed by RP-HPLC on a Nexera LC-40 machine (Shimadzu, Japan) equipped with a ReproSil-Pur Basic C-18 column (150 mm x 4.6 mm, 5 μm; Dr. Maisch HPLC GmbH, Ammerbuch, Germany) connected with SPD-M20A photo diode array detector (Shimadzu, Japan). For 4CP determination, the mobile phase consisted of acetonitrile and methanol, 1% acetic acid (20:20:60 v/v/v), for IOX - acetonitrile and 1% acetic acid (5:95 v/v), accordingly. Separation was carried out using an isocratic flow of 1 mL/min, the wavelengths were set at 272 nm and 245 nm for 4CP and IOX detection, respectively. To calculate the concentration of the tested compounds in the samples, calibration curves of the area under the curve versus concentration were used. The calibration curve for 4CP was described by the equation y = 689.28x, and for IOX, y=15481x.

### Statistical analysis

For each experiment, the distribution of results has been checked using the Shapiro-Wilk test. FAME profiles have been compared using analysis of variance (ANOVA) and Tukey’s post-hoc test. Additionally, principal component analysis (PCoA) has been conducted, and statistical significance has been tested using multidimensional permutation (PERMANOVA). Statistical analysis of bisphenol removal from culture media was conducted using Student’s t-test, while removal of 4CP and IOX was analyzed using ANOVA and Tukey’s post-hoc tests.

Normality test, Student’s t-test and ANOVA analyses have been conducted using Jamovi software v 2.3.2 (https://www.jamovi.org). PCoA and PERMANOVA analyses have been conducted in R v 4.1.0 using the vegan package v 2.5–7 (Oksanen et al. 2025).

## Results

### Isolation and growth of strain MW-W600-9^T^

The strain MW-W600-9^T^ was isolated from collective shaft coal mine waters on PDA medium. Strain colonies showed typical actinomyces-like morphology. It grew as a compact orange colony, over time, with a white surface. Microscopic investigation showed branching morphology of Gram-stain-positive microorganisms and formation of the pseudomycelium (supplementary materials Fig. S1).

Growth of the strain on several microbiological media has been evaluated (Table 1). The strain was growing on all tested media, although at different rates. The fastest growth was observed on PDA, ISP1, ISP2, Nutrient Agar, BHI Agar, Blood Agar, Columbia Blood Agar, Medium 79, TGTS medium, Sabouraud Agar, Löwenstein-Jensen Agar media, while the slowest growth was on AGS, ISP3, ISP4, ISP9 and MSMA-C2 media. The strain grew in aerobic conditions; no growth was observed in anaerobic conditions.

**Table 1:** Growth of the strain MW-W600-9^T^ on different culture media

Medium	Growth	Substrate mycelium	Aerial mycelium
PDA	**+++**	colorless	orange with white surface over time
AGS	**+**	colorless	orange with white surface over time
ISP1	**+++**	colorless	orange with white surface over time
ISP2	**+++**	colorless	orange with white surface over time
ISP3	**+**	colorless	colorless with white surface over time
ISP4	**+**	colorless	colorless with white surface over time
ISP9	**+**	colorless	colorless with white surface over time
AIA	**++**	colorless	orange with white surface over time
Nutrient Agar	**+++**	colorless	cream-yellow with white surface over time
BHI Agar	**+++**	colorless	orange with white surface over time
Blood Agar	**+++**	ND	cream-yellow with white surface over time
Columbia Blood Agar	**+++**	ND	cream-yellow with white surface over time
Medium 79	**+++**	colorless	salmon pink with white surface over time
TGTS Medium	**+++**	colorless	orange with white surface over time
Sabouraud Agar	**+++**	colorless	salmon pink with white surface over time
Löwenstein-Jensen Agar	**+++**	colorless	yellow-orange with white surface over time
MSMA-C2	**+**	colorless	colorless with white surface over time

Note: “+++” - superior growth, “++” - moderate growth, “+” - weak growth; *PDA* Potato dextrose agar, *AGS* Arginine, glycerol, salts medium, *ISP* International Streptomyces Project media, *AIA* Actinobacteria Isolation Agar, *BHI* Brain Hearth Infusion, *TGTS* thioglycolate-trypticasein soy agar, *MSMA-C2* hard-coal supplemented minimal salt media agar; *ND* Not determined

Further, the growth of the strain at different temperatures (Table 2), pH and salt concentration (Table 3) was examined. The strain was capable of growth at 25 °C when on PDA and at both 25 °C and 37 °C when cultured on ISP2 medium. Furthermore, it was able to survive 7 days at 4 °C and 8 °C. On the other hand, strain was not able to grow or survive at 42 °C and 45 °C after 7 days of incubation on either of the media. Strain MW-W600-9^T^ was able to grow at NaCl concentrations in the range from 0% to 4%, with the best growth at 0.5%, while at 4% the growth was minimal. For pH, the strain grew from pH 5.0 to pH 9.0. The best growth was at pH 7.0. The strain also tolerated basic pH values (pH 8.0 and pH 9.0) better than slightly acidic (pH 5.0).

**Table 2:** Growth of the strain MW-W600-9^T^ at different temperatures

		Temperature					
Medium		4 °C	8 °C	25 °C	37 °C	42 °C	45 °C
PDA	Growth	-	-	+	-	-	-
	Viability	+	+	+	+	-	-
ISP2	Growth	-	-	+	+	-	-
	Viability	+	+	+	+	-	-

Note: “+” - growth, “-” - no growth

**Table 3:** Growth of the strain MW-W600-9^T^ at different salinity and pH

Salinity (NaCl concentration)														
0%	0.5%		1%		1.5%		2%	4%		6%		8%		10%
++	+++		++		++		++	+		-		-		-

pH														
4		5		6		7			8		9		10	
-		+		**++**		+++			++		++		+	

Note: “+++” - superior growth, “++” – moderate growth, “+” – weak growth, “-” – no growth

### Genome sequencing and phylogeny

The sequencing of the 16S rRNA gene of the strain MW-W600-9^T^ and search against NCBI’s 16S/ITS database showed that the strain belongs to the genus *Nocardia*. The partial 16S rRNA gene sequence has the highest similarity to sequences from *N. asteroides* (99.76% and 99.60%), *N. neocaledoniensis* (99.36% and 99.29%) and *N. rhizosphaerihabitans* (99.29%). The phylogenetic tree constructed from the alignment of 16S rRNA gene sequences showed that the strain belongs to a branch with afore mentioned as well as several other *Nocardia* species (supplementary materials Fig. S2). Importantly, the sequence of strain MW-W600-9^T^ is located at the root of the clade and is branching out early from the rest of the species. As the 16S rRNA gene comparison might not be enough to clearly establish the taxonomical status of the new isolates, especially in the groups where a large number of species are recognized, we have performed analysis based on whole-genome sequencing. This approach is further supported by the fact that the whole genome sequences are available for the majority of closely related species, including type strains of these species.

**Fig. 1:**
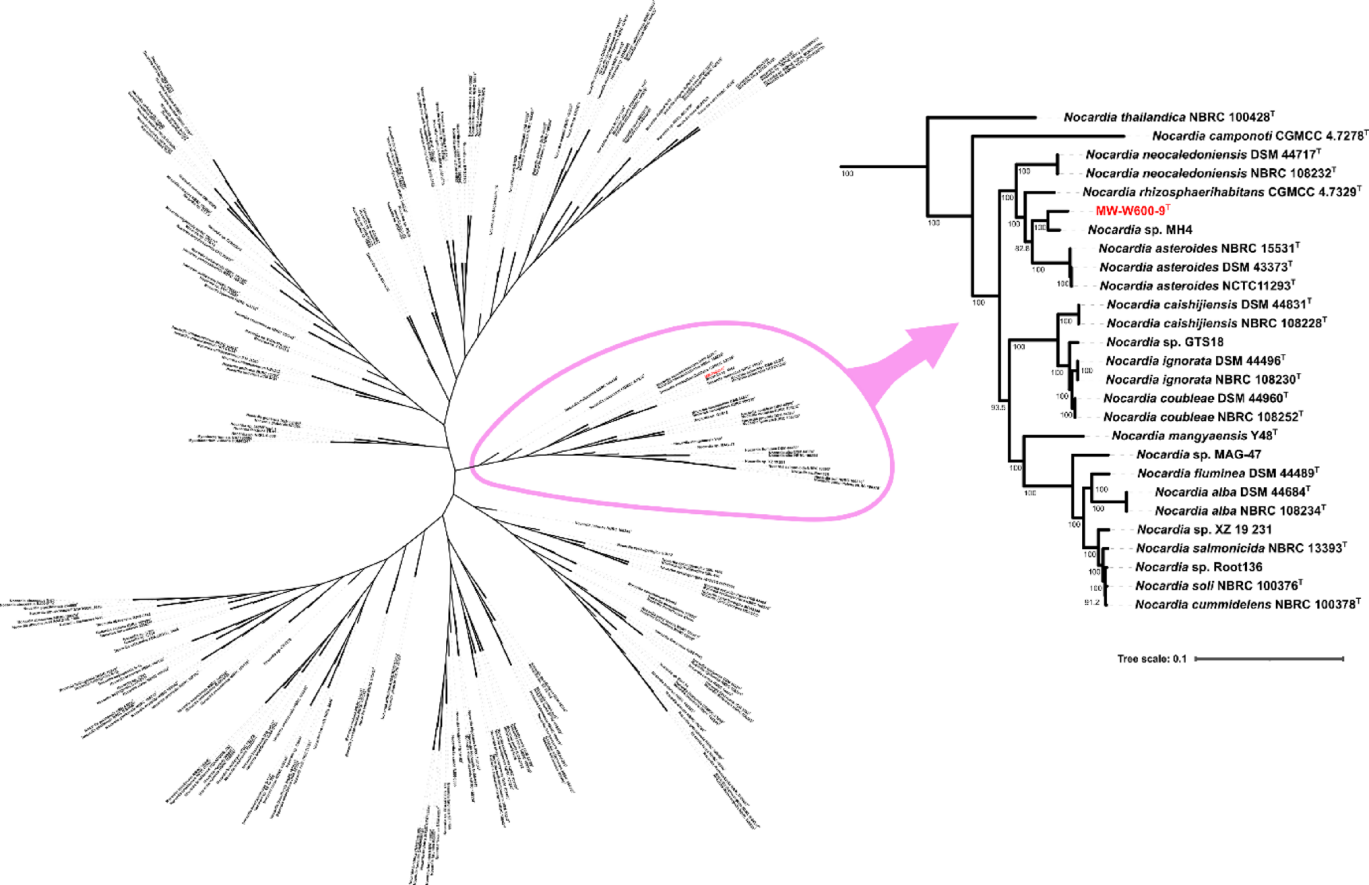
Maximum-likelihood phylogenomic tree calculated based on core proteome alignment of 257 genomes of *Nocardia* and 2 genomes of *Mycobacterium* used as an outgroup. The clade with the strain MW-W600-9^T^ is shown as a phylogram, with the position of the strain marked in red. Numbers on the branches indicate bootstrap values (as % of 1000 repeats)

The whole genome sequencing of the strain MW-W600-9^T^ resulted in assembly of 7,709,572 bp in size, 68.79% of G + C nucleotides with 98.4% completeness (Table 4). Depending on the used tool, the genome contains from 6557 to 7551 CDS. The draft genome sequence was used to characterize genome sequence-related parameters, including average nucleotide identity (ANIb), digital DNA–DNA hybridization (dDDH), and the diversity of secondary metabolite biosynthetic gene clusters. Both dDDH, as well as ANIb showed that the strain belongs to the novel species of the *Nocardia* genus, as both dDDH and ANIb are below the reference level (Table 5 and supplementary materials Tables S1 and S2). The most similar strain to the isolate was *Nocardia* sp. MH4 (51.1% and 92.28% for dDDH and ANIb, respectively). From type strains, two of the most similar strains were *N. rhizosphaerihabitans* CGMCC 4.7329^T^ (37.3% and 87.66% for dDDH and ANIb, respectively) and *N. asteroides* DSM 43373^T^ (34.8% and 86.79% for dDDH and ANIb, respectively), which is in accordance with analysis of 16S rRNA gene against 16S/ITS NCBI’s database. Along with the dDDH pairwise trees calculation based on 16S rRNA sequence and whole genome sequences of strains included in comparison have been conducted (supplementary materials Fig. S3). Results from both trees showed that the strain MW-W600-9^T^ forms a separate branch in a clade with *Nocardia* sp. MH4, *N. asteroides* DSM 43373^T^, *N. rhizosphaerihabitans* CGMCC 4.7329^T^ and *N. neocaledoniensis* DSM 44717^T^, although the exact topography of the trees showed slight differences. A maximum-likelihood phylogenetic tree based on the core proteome (Fig. 1) confirmed that the strain MW-W600-9^T^ forms a clade with *Nocardia* sp. MH4 to which it is most related, and *N. asteroides*,* N. rhizosphearihabitans* and *N. neocaledoniensis* species. Like pairwise trees and opposite to the 16S rRNA gene maximum-likelihood tree, the core proteome-based tree showed that the strain is not at the root of the clade. The analysis indicated that both strains, MW-W600-9^T^ and MH4, are related to each other and share a common ancestor with *N. asteroides*. This subclade shares ancestry with *N. rhizosphaerihabitans* and more distantly with *N. neocaledoniensis.* All together, the results strongly suggest that strain MW-W600-9^T^ is a new species of *Nocardia*, although the exact relation to the evolutionary closest species is elusive. Further chemotaxonomic analyses were conducted to establish its taxonomic status.

**Table 4:** The whole genome sequencing data and general genomic features of *Nocardia* sp. MW-W600-9^T^

Assembly details	
Contigs	2
G + C content	68.79%
Contig L50	1
Contig N50	7,602,475 bp
Contig N99	7,602,475 bp
Genome Length	7,709,572 bp
Coverage	34.5
Completeness	98.4%
CDS	7551 (RAST-Tk); 7213 (BlastKoala); 6557 (eggNOG-mapper)
tRNA	57
rRNA	15

**Table 5:** Comparison of MW-W600-9^T^ to phylogenetically closely related strains

Strain	dDDH (d4, [C.I.], %)	G + C difference, %	ANIb (%)	Aligned (%)	Aligned (bp)
*Nocardia* sp. MH4	51.1 [48.5–53.8]	0.78	92.28	74.57	5,749,245
*N. rhizosphaerihabitans* CGMCC 4.7329^T^	37.3 [34.9–39.9]	0.37	87.66	68.23	5,260,028
*N. asteroides* DSM 43373^T^	34.8 [32.4–37.3]	1.14	86.79	67.21	5,181,832
*N. neocaledoniensis* DSM 44717^T^	31.9 [29.5–34.4]	0.88	85.18	63.99	4,933,696
*N. mangayaensis* Y48^T^	30.5 [28.1–33.0]	0.7	83.93	60.30	4,648,972
*N. fluminea* DSM 44489^T^	30.0 [27.6–32.5]	1.3	84.06	63.76	4,915,934
*N. salmonicida* NBRC 13393^T^	29.8 [27.4–32.3]	1.76	83.99	65.39	5,041,567
*N. soli* NBRC 136^T^	28.8 [26.4–31.3]	1.74	83.13	60.79	4,686,837
*Nocardia* sp. Root136	28.6 [26.2–31.1]	1.47	83.11	60.36	4,653,837
*Nocardia* sp. XZ19 231	28.5 [26.1–31.0]	1.74	83.03	59.32	4,573,601
*Nocardia* sp. MAG-47	28.3 [25.9–30.8]	1.3	82.69	52.55	4,051,227
*N. alba* DSM 44684^T^	28.0 [25.7–30.5]	1.12	82.59	56.79	4,378,402
*N. ignorata* DSM 44496^T^	27.9 [25.6–30.4]	1.07	82.56	59.09	4,555,542
*N. coubleae* DSM 44960^T^	27.8 [25.4–30.3]	0.85	82.46	58.56	4,514,898
*N. caishijensis* DSM 44831^T^	27.6 [25.2–30.1]	0.56	82.09	53.06	4,090,750
*Nocardia* sp. GTS18	27.5 [25.2–30.0]	0.95	81.11	45.85	3,535,136

Values of digital DNA-DNA hybridization (dDDH) from pairwise comparison of genomes using TYGS server and average nucleotide identity using BLAST (ANIb)

Note: C.I. – confidence interval, bp – base pairs

### Metabolic and growth profiling

Metabolic and growth profiles of the strain MW-W600-9^T^ and two type strains from neighboring species, *N. rhizosphaerihabitans* DSM 101726^T^ (same as CGMCC 4.7329^T^) and *N. asteroides* DSM 43757^T^ (same as DSM 43373^T^) were obtained using the GEN III MicroPlate™ assay (supplementary materials Fig. S4). All three strains are catalase-positive and able to grow on L-histidine, β-hydroxybutyrate, acetoacetate, acetate and pyruvate as a source of carbon. Aesculin is hydrolyzed. They show growth in 1% sodium lactate, guanidine chloride, sodium bromate, potassium tellurite and Tween 40. All strains show tolerance to NaCl concentration between 1 and 4%, which confirms results from salinity tolerance by the strain MW-W600-9^T^. On GENIII plates, only *N. asteroides* DSM 43757^T^ shows growth at pH 5.0, while all of the strains are able to grow at pH 6.0. The strains show resistance to rifampicin, lincomycin, fusidic acid, nalidixic acid and aztreonam. The conditions for which strains differ in growth are presented in Table 6. The strain MW-W600-9^T^ is the only strain of these three that can grow on D-salicin, methyl pyruvate and α-keto-glutaric acid. It shares some metabolic similarities with either one of the related strains used for comparison. Both strains, MW-W600-9^T^ and *N. rhizosphaerihabitans* DSM 101276^T^ can grow on N-acetyl-β-D-glucosamine, D-fructose and citric acid. While no growth was observed for either of the two in the presence of D-fructose-6-phosphate, formic acid or sodium butyrate and they showed no resistance to troleandomycin. At the same time, similarities in metabolic and growth conditions between the isolate and *N. asteroides* DSM 43757^T^ are the ability to grow on glycerol and α-keto-butyric acid, as well as no resistance to vancomycin and p-hydroxy-phenylacetic acid.

**Table 6:** The metabolic profile and growth conditions for *Nocardia* sp. MW-W600-9^T^ and related type strains

Condition	MW-W600-9^T^	DSM 101726^T^	DSM 43757^T^
pH 5	-	-	+
D-Salicin	+	-	-
N-Acetyl-β-D-Glucosamine	+	+	-
D-Fructose	+	+	-
Glycerol	+	-	+
D-Fructose-6-Phosphate	-	-	+
Troleandomycin	-	-	+
Vancomycin	-	+	-
p-Hydroxy-Phenylacetic Acid	-	+	-
Methyl Pyruvate	+	-	-
Citric Acid	+	+	-
α-Keto-Glutaric Acid	+	-	-
α-Keto-Butyric Acid	+	-	+
Formic Acid	-	-	+
Sodium Butyrate	-	-	+

The conditions for which a difference between strains was observed are shown

### Comparative chemotaxonomy

**Fig. 2:**
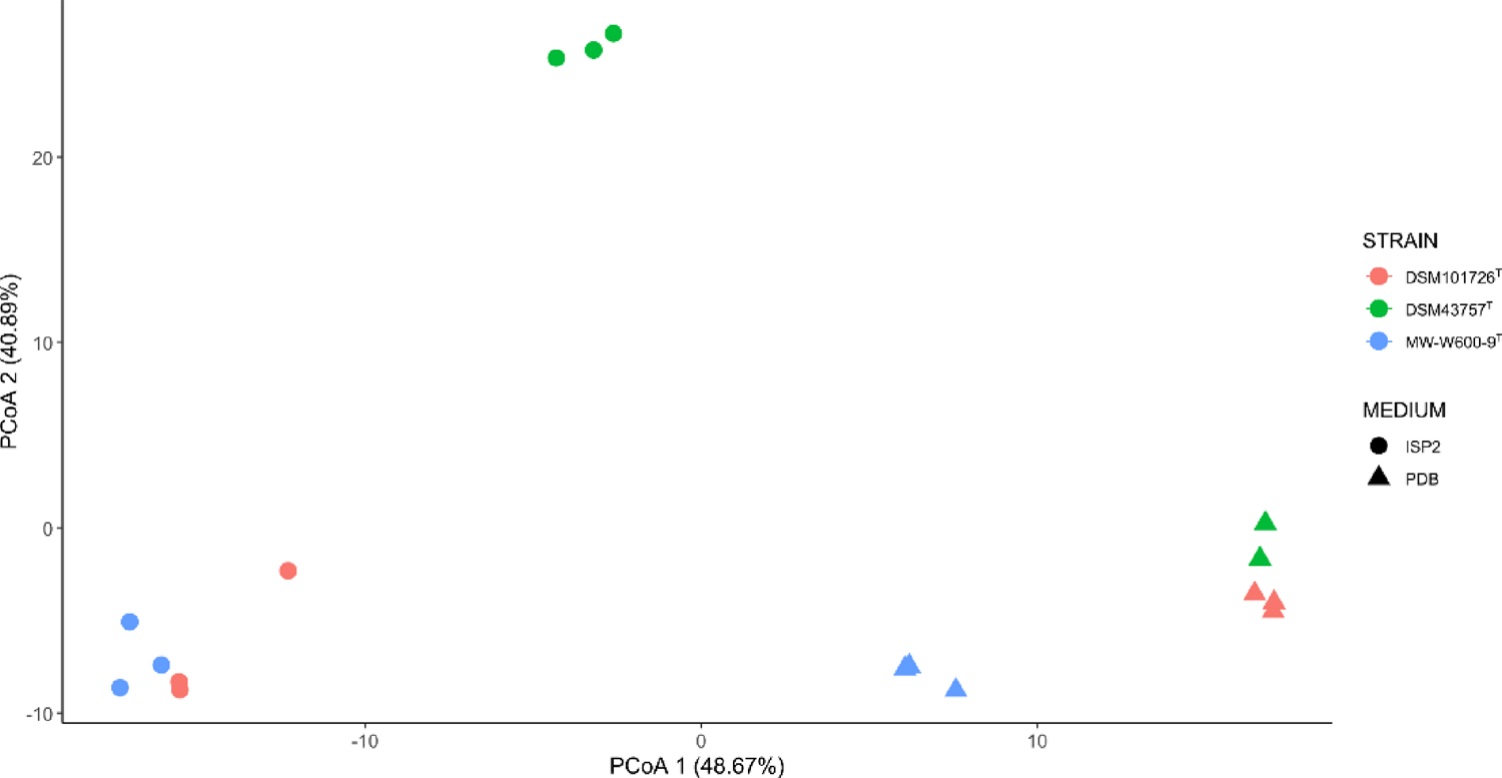
Principal Coordinate Analysis of fatty acids methylated ester profiles of strains MW-W600-9^T^, *N. rhizosphaerihabitans* DSM 101726^T^ and *N. asteroides* DSM 43757^T^. Each point represents a biological repeat. Differences between profiles from all conditions were statistically significant (PERMANOVA, *p* < 0.05)

FAME profiles of the three strains grown on PDB and ISP2 media were compared. Detailed data for detected fatty acids and their relative abundance are presented in the supplementary materials Table S3. The strain shows a similar ratio of saturated and unsaturated fatty acids on both media, with an increased fraction of saturated fatty acid when they are cultivated on ISP2 in comparison to PDB medium (supplementary materials Fig. S5). Dominant saturated and unsaturated fatty acids are 16:0 and 18:1, respectively (supplementary materials Table S3). PCoA analysis of FAME profiles (Fig. 2) shows that strains differ in composition of fatty acids, with the strain MW-W600-9^T^ profile being more similar to *N. rhizosphaerihabitans* DSM 101726^T^ on ISP2 medium, while on PDB the isolated profile was more distant from the *N. rhizosphaerihabitans* DSM 101726^T^ and *N. asteroides* DSM 43757^T^ profiles.

**Fig. 3:**
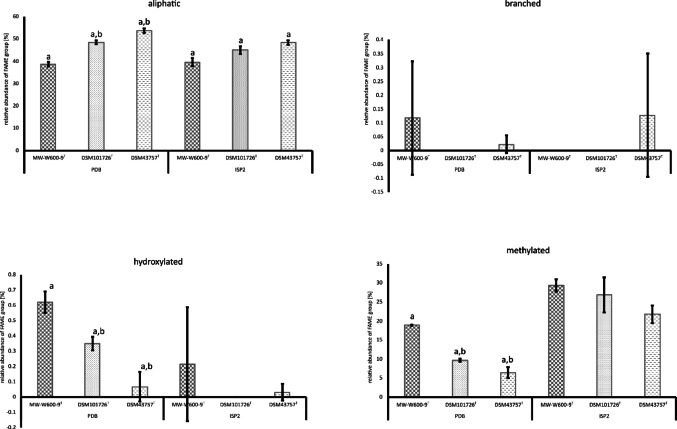
Plots showing the relative contribution of different classes of fatty acids to the FAME profiles of the strains MW-W600-9^T^, *N. rhizosphaerihabitans* DSM 101726^T^, and *N. asteroides* DSM 43757^T^. Means of three independent biological repeats are shown; error bars represent standard deviations. Statistically significant differences are labelled ‘a’ – differences between the MW-W600-9^T^ and other strains, and ‘b’ – differences between two other *Nocardia* strains (ANOVA with Tukey’s post-hoc test, *p* < 0.05)

In all three strains, acids 17:0, 10Me and 18:0 10Me are present. The contribution of aliphatic, branched, hydroxylated and methylated fatty acid differs between strains (Fig. 3). The strain MW-W600-9^T^ has the lowest fraction of aliphatic fatty acids (38.72 ± 1.05% on PDB and 39.67 ± 1.85% on ISP2) compared to DSM 101726^T^ (48.52 ± 0.83% on PDB and 45.02 ± 1.84% on ISP2) and DSM 43757^T^ (53.69 ± 0.96% on PDB and 48.43 ± 0.99% on ISP2). The contribution of this fraction is significantly different between strains on PDB medium and between the strain MW-W600-9^T^ and two other strains on ISP2 medium. Branched fatty acids are present only in the strain DSM 43757^T^ on ISP2 medium and in the isolated strain and strain DSM 43757^T^ on PDB medium. But in both cases, their contribution was small and large differences between repeats were observed. For hydroxylated and methylated fatty acids, statistically significant differences between strains are present on PDB medium but not on ISP2 medium (Fig. 3). Both fractions were the highest in the strain MW-W600-9^T^ (0.62 ± 0.07% and 18.95 ± 0.13% for hydroxylated and methylated fatty acids respectively), while the lowest for strain DSM 43757^T^ (0.07 ± 0.09% and 6.47 ± 1.4% for hydroxylated and methylated fatty acids respectively). The strain MW-W600-9^T^ is distinguished from the other strains by the presence of 19:0 *iso*, 15:1 *iso* F, 17:1 *iso* ω9c, 19:1 *iso* I acids and lack of 12:0 and 20:2 ω6,9c acids on PDB medium, as well as the presence of 17:0 *iso* 3OH, 19:0 10Me, 17:1 ω8c, 18:1 *iso* H acids on ISP2 medium.

The analysis of mycolic acid using TLC has revealed that the strain MW-W600-9^T^ contains acid with the same mobility as nocardiomycolic acids and did not differ from strains used for comparison (supplementary materials Fig. S6). Furthermore, MALDI-ToF mass spectra of mycolic acids isolated from strains MW-W600-9^T^, *N. rhizospaerihabitans* DSM 101726^T^, and *N. asteroides* DSM 43757^T^ show the same major ions (supplementary materials Fig. S7). The cluster peaks of mycolic acids differ in 28 mass units and cover mycolates with 52–60 carbon atoms.

The analysis of polar lipids revealed that the strain MW-W600-9^T^ contains several components, including phosphatidylethanolamine, diphosphatidylglycerol, phosphatidylinositol, and phosphatidylinositol mannosides. Interestingly, analysis detected the presence of an unknown lipid in the strain that is not present in the other strains (supplementary materials Fig. S8). A comparative analysis with DSM 101726^T^ and DSM 43757^T^ strains using 2D-TLC showed that strains share the same profiles for both phospholipids and glycolipids, except *N. rhizosphaerihabitants* DSM 101726^T^, where an additional lipid spot was detected for total lipids 2D-TLC analysis (supplementary materials Fig. S6).

Whole-cell wall hydrolysate of the strain MW-W600-9^T^ showed it contains meso-diaminopimelic acid in peptidoglycan, with arabinose and galactose identified as the primary diagnostic sugars. Similar profiles were present in two other *Nocardia* strains used for comparison (supplementary materials Fig. S9). N-glycosylated muramic acid was not detected in the peptidoglycan of the isolate.

### MALDI-ToF MS protein profiling

Strains MW-W600-9^T^, *N. asteroides* DSM 43757^T^, and *N. rhizosphaerihabitans* DSM 101726^T^ were grown under the same conditions and were identified by protein profiling using a MALDI-TOF MS Biotyper (Table 7). The protein spectra of *N. asteroides* were included in the Biotyper database used for identification, and these strains were correctly identified, in contrast to those of *N. rhizosphaerihabitans* and the strain MW-W600-9^T^, which were not reliably identified with a score below 1.7. The protein mass spectra generated for the strain MW-W600-9^T^ were distinct from *N. asteroides* DSM 43757^T^ and *N. rhizosphaerihabitans* DSM 101726^T^; the strain MW-W600-9^T^ protein profile was more similar to the latter (supplementary materials Fig. S10).

**Table 7:** MALDI-ToF Biotyper identification of the strains MW-W600-9^T^, *N. rhizosphaerihabitans* DSM 101726^T^, and *N. asteroides* DSM 43757^T^ in the Bruker Biotyper database

Strain	Condition^a^	Organism	Score Value^b^
DSM 43757^T^	79L_3 days 25 °C	*N. asteroides*	1.742
DSM 43757^T^	BL_4 days 25 °C	*N. asteroides*	2.174
DSM 101726^T^	79L_4 days 25 °C	NR	1.433
DSM 101726^T^	BL_4 days 25 °C	NR	1.328
MW-W600-9^T^	79L_3 days 25 °C	NR/*N. asteroides*	1.480
MW-W600-9^T^	BL_3 days 25 °C	NR	1.384

^a^*Nocardia* species were grown aerobically on liquid yeast extract glucose agar, medium 79 (Paściak et al. 2014) (79 L), and blood agar (BL) for 3–4 days at 25 °C. The samples were prepared by the formic acid-acetonitrile extraction method

^b^Score values determined by MALDI Biotyper: <1.7 – identification not reliable (NR), 1.7–2.0 – probable genus identification, 2.0–2.3 – secure genus identification and probable species identification, > 2.3 – highly probable species identification

### Biosynthetic potential and antimicrobial activity

**Fig. 4:**
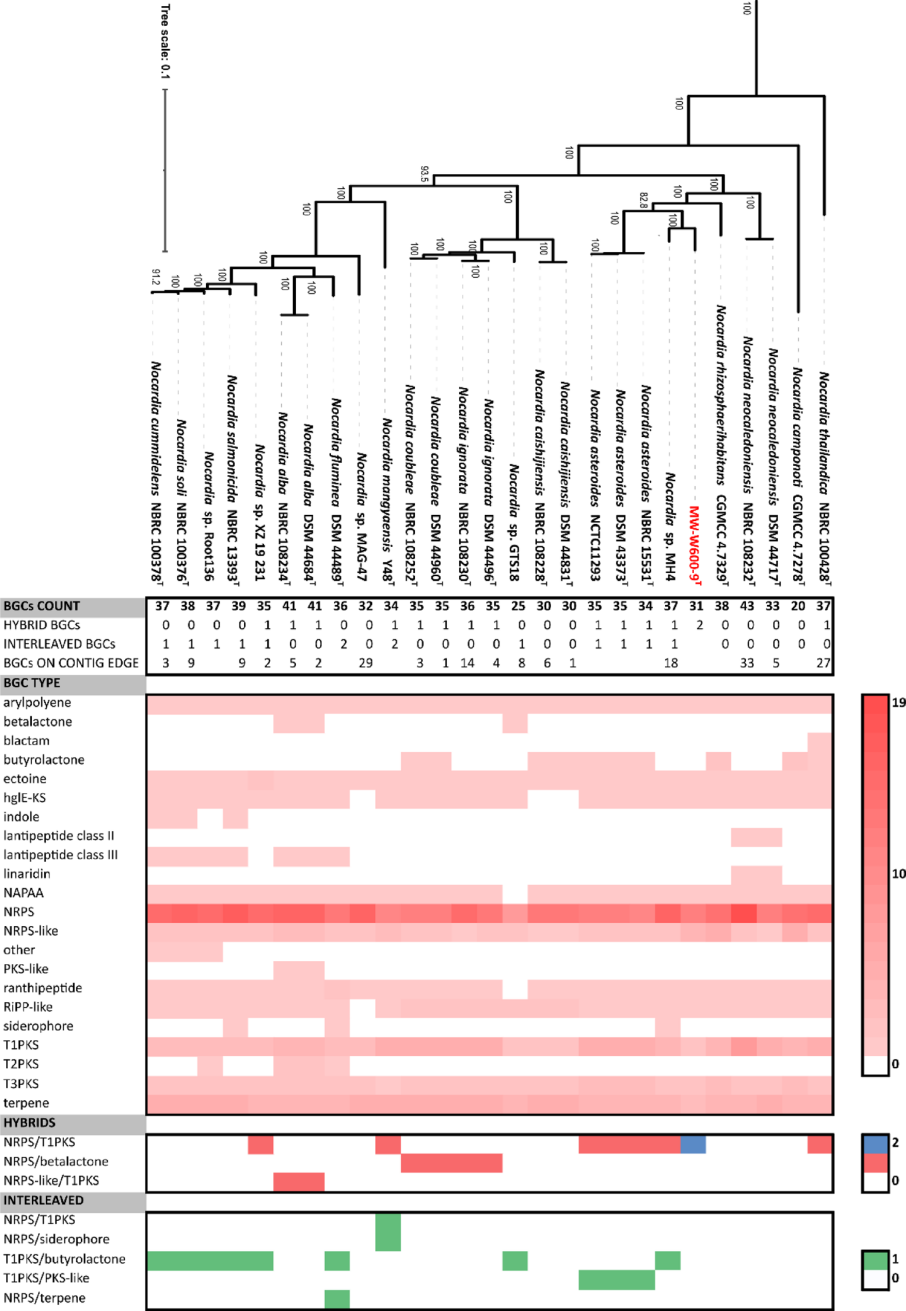
Heat map of putative BGCs detected in genomes of the strain MW-W600-9^T^ and taxonomically related strains. The taxonomic relationship between the compared strains is shown on a maximum-likelihood tree based on the core proteome; numbers on branches show bootstrap values (in % of 1000 repeats). The strain M-W600-9^T^ is marked in red. NRPS – non-ribosomal peptide synthetases, T1PKS, T2PKS, T3PKS – type 1, 2, 3 polyketide synthetases, hgIK-KS – heterocyst glycolipid synthetase-like polyketide synthase, NAPAA - non-alpha poly-amino acids like ε-polylysin, RiPP – ribosomally synthesized post-translationally modified peptide, blactam – β-lactam

Potential for production of secondary metabolites by the strain MW-W600-9^T^ was examined. Thirty-one putative BGCs in 22 regions were detected in the genome of the isolate (Fig. 4; Table 8). The number of the strain’s putative BGCs is slightly below the average for closely related *Nocardia* strains (34.78 BGCs per genome). However, no BGCs were detected on the contig edge, while this was the case for some of the evolutionarily related strains (Fig. 4), so the real number of BGCs in their genomes is probably smaller. The most enriched type of BGCs in the genome of the strain MW-W600-9^T^, making 38.71% (12 BGCs), were non-ribosomal peptide synthetases (NRPS), then NRPS-like BGCs (12.9%; 4 BGCs), and the third were terpenes (9.68%, 3 BGCs). Other detected classes included arylpolyene, NAPAA, ranthipeptides, RiPP-like BGCs, type 1 polyketide synthetases (T1PKS) and type 3 polyketide synthetases (T3PKS). Two hybrid BGCs were also detected, both being NRPS/T1PKS hybrids (Fig. 4). Only 4 of 22 regions contain clusters with similarity to known clusters above 20%, with just 2 with similarity over 30% (ectoine cluster in region 1.1–100% similarity and NRPS cluster in region 1.15–87% similarity to nocobactin NA). Twelve regions had less than 10% similarity to known clusters, and 3 of them had no similarity (one terpene, T1PKS and arylpolyene clusters) (Table 8).

**Table 8:** *Nocardia* sp. MW-W600-9^T^ putative biosynthetic gene clusters

BGC’s region	BGC’s type	Most similar known cluster	Similarity (%)
Region 1.1	ectoine	ectoine	100
Region 1.2	NRPS	calicheamicin	2
Region 1.3	NRPS-like, T3PKS	merochlorin A/B/C/D, dichloro-merochlorin B, deschloro-merochlorin A/B, isochloro-merochlorin B	12
Region 1.4	hgEI-KS	pradimicin-A	7
Region 1.5	terpene	-	-
Region 1.6	NRPS	maklamicin	4
Region 1.7	NRPS	atratumycin	7
Region 1.8	T1PKS	vicenistatin	20
Region 1.9	RiPP-like, NRPS, ranthipeptide	borrelidin	4
Region 1.10	NRPS, T1PKS	cryptophycin-327	25
Region 1.11	T1PKS	-	-
Region 1.12	NAPAA	ulleungmycin	5
Region 1.13	NRPS	mycobactin	30
Region 1.14	NRPS, terpene	atratumycin	7
Region 1.15	NRPS	nocobactin NA	87
Region 1.16	NRPS, NRPS-like	enduracidin	10
Region 1.17	terpene	carotenoid	27
Region 1.18	arylpolyene	-	-
Region 1.19	T1PKS, NRPS	foxicins a-d	4
Region 1.20	T3PKS	s56-p1	3
Region 1.21	NRPS	pentalenolactone	15
Region 1.22	NRPS-like	chloromyxamide	10

Biological activity against bacteria, yeast and mycelial fungi has been tested. However, in the culture conditions used for the experiment, the strain MW-W600-9^T^ did not show any of these activities (data not shown).

### The strain MW-W600-9^T^ potential for the removal of aromatic compounds from culture

Functional annotation classified 34% (2455 of 7213 protein coding sequences) and 40% (2618 of 6040 protein coding sequences) of putative protein coding sequences using BlastKoala and eggNOG-mapper, respectively. Annotations were analyzed for the presence of biodegradation genes. A total of nine genes potentially involved in the initial steps of degradation of aromatic xenobiotic compounds have been detected (Table 9). Eight of the genes belong to oxygenases, three monooxygenases, *cyp125*, *cyp199A2* and *pobA*, and six dioxygenases, *catA*, *hsaC_1*, *hcaD*, *chqB* and *hmgA*. The last detected gene is haloalkane dehalogenase, *dhaA_1*, suggesting the strain’s potential to degrade halogenated compounds.

**Table 9:** List of genes potentially involved in the cleavage of ring structure in aromatic organic compounds detected in *Nocardia* sp. MW-W600-9^T^

Gene	Enzyme	Detection software
*cyp125*	cholest-4-en-3-one 2,6-monooxygenase	eggNOG/BlastKoala
*dhaA1*	haloalkane dehalogenase	eggNOG/BlastKoala
*catA*	catechol 1,2-dioxygenase	eggNOG/BlastKoala
*cyp199A2*	4-methoxybenzoate monooxygenase	BlastKoala
*pobA*	p-hydroxybenzoate 3-monooxygenase	eggNOG/BlastKoala
*hsaC1*	3,4-dihydroxy-9,10-secoandrosta-1,3,5(10)-triene-9,17-dione 4,5-dioxygenase	eggNOG/BlastKoala
*hcaD*	3-phenylpropionate/trans-cinnamate dioxygenase ferredoxin reductase component	eggNOG/BlastKoala
*chqB*	hydroxyquinol 1,2-dioxygenase	eggNOG/BlastKoala
*hmgA*	homogentisate 1,2-dioxygenase	eggNOG/BlastKoala

**Fig. 5:**
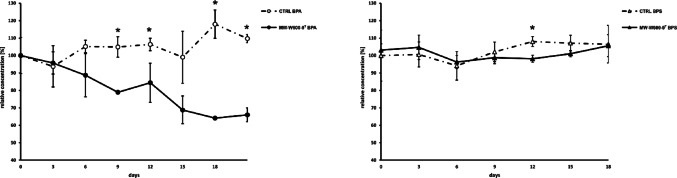
Plots showing relative concentrations of BPA (left, circles) and BPS (right, triangles) in control (dashed line, open symbol) and the strain MW-W600-9^T^ culture (solid line, filled symbols). Points represent the means of three independent repeats; error bars represent the standard deviation. Statistically significant differences between the strain MW-W600-9^T^ and control cultures have been labelled with an asterisk (Student’s t-test, *p* < 0.05)

The ability of the strain MW-W600-9^T^ to remove aromatic xenobiotics from culture medium has been examined for two bisphenols and aromatic haloorganic compounds − 4CP and IOX. Because the compounds themselves might not be sufficient sources of carbon and energy, experiments were conducted in R2A medium. Results from the removal of bisphenol are shown in Fig. 5. The concentration of BPA has been significantly lower compared to the control from the 9th day of culture until the end of the experiment, except on day 15 due to large variations in measurements in control samples. The concentration has been dropping continuously during the course of the experiment, suggesting degradation of the compound. After 21 days, there was a 43.73% reduction in BPA concentration. On the other hand, for BPS, no significant differences between the strain culture and control were observed. The exception was day 12, when the difference in concentration was significant, but later the BPS concentration in the strain MW-W600-9^T^ culture increased again, leading to no significant difference in comparison to the control. That might be explained by the initial accumulation of BPS on the surface of the cells and subsequent release during the course of the experiment (Fig. 5).

**Fig. 6:**
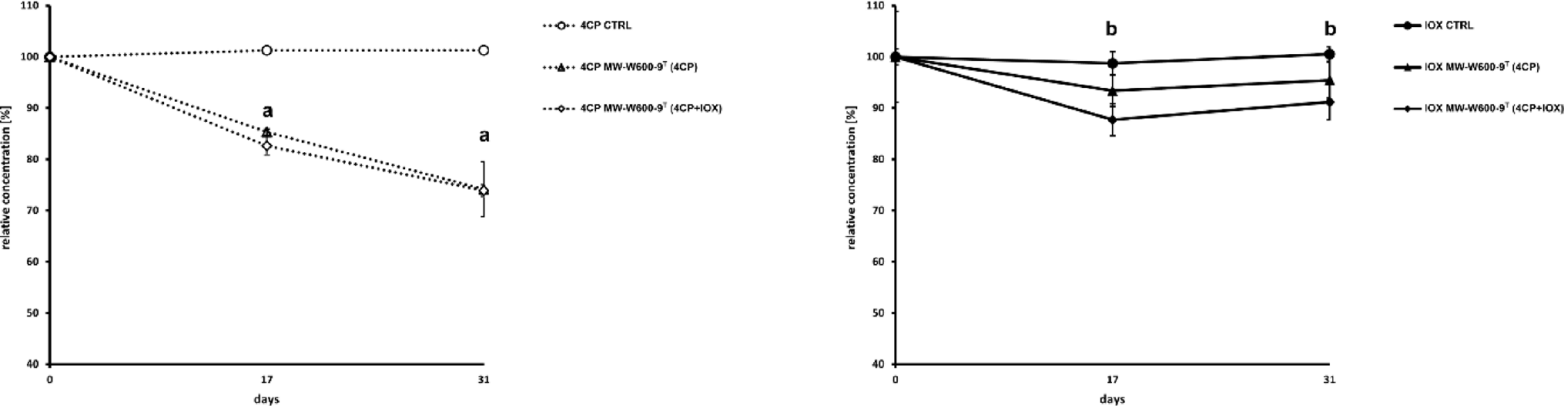
Plots showing relative concentrations of 4CP (left, open symbols, dashed lines) and IOX (right, filled symbols, solid lines) in control (circle) and cultures of the strain MW-W600-9^T^ cultures with 4CP (triangle) or IOX (diamond). Points represent the means of three independent repeats; error bars represent the standard deviation. Statistically significant differences have been labelled with ‘a’ – difference between both cultures of the strain MW-W600-9^T^ cultures and control culture, ‘b’ – difference between 4CP + IOX culture of the strain MW-W600-9^T^ and control culture (Student’s t-test, *p* < 0.05)

4CP and IOX removal experiments have been conducted for 31 days, as these compounds are expected to be more resistant to degradation. Additionally, a culture with both xenobiotics has been prepered where 4CP could function as a stimulant of dehalogenase involved in the degradation of IOX. The results are presented in Fig. 6. For 4CP, there was a statistically significant difference in concentration between control and both 4CP alone and 4CP + IOX cultures. 4CP concentration dropped steadily in both cultures of the strain MW-W600-9^T^ during the course of the experiment, leading to 27.12% and 27.38% removal in 4CP and 4CP + IOX cultures, respectively. At the same time, IOX concentration showed a significant difference compared to the control in 4CP + IOX culture but not in IOX culture at days 17 and 31. However, the difference in IOX concentration did not differ significantly between IOX and IOX+4CP cultures at any time point. Furthermore, in each culture of the strain MW-W600-9^T^, an increase in IOX concentration on day 31 was observed in comparison to day 17, suggesting absorption of the compound by the cells and later release to the medium over the course of the culture (Fig. 6). The removal of IOX in the IOX+4CP culture compared to the control at the end of the experiments was 10.12%.

## Discussion

The study describes a new species, *Nocardia fodinahabitans* sp. nov., with MW-W600-9^T^ being a type strain. The strain was isolated from underground coal mine waters and has the ability to degrade aromatic organic compounds, including xenobiotics. The work is a first report of the degradation of bisphenol by a member of *Nocardia* and suggests co-metabolic degradation of environmentally persistent haloorganic compound, iohexol. This points to potential use of the strain in bioremediation applications alone or as a part of a consortium. Furthermore, the strain has 12 putative biosynthetic gene clusters with low or no similarity to known compounds, highlighting its potential as a source of novel molecules of importance for biotechnology. Future work will further explore strain biodegradation capabilities and will include confirmation and characterization of degradation pathways, optimization of the process and evaluation of the use of the strain as part of bioremediation consortia. Additionally, identification and characterization of products of novel putative biosynthetic gene clusters should be carried out.

Extreme and previously unexplored environments can be a source of valuable microorganisms for a range of biotechnological applications. This includes underground environments (Siupka et al. 2021). Coal mines are one such environment, and previously, microorganisms with biotechnological potential have been isolated from them (Kim et al. 2019; Siupka et al. 2021). Organisms living in underground environments can represent novel species, but even when this is not the case, they can have unique features distinguishing them from their counterparts isolated from the soil of surface waters (Gosse et al. 2019). In the current work, we have isolated a new bacterial strain from underground coal mine water. Strain’s growth phenotype and formation of branching pseudomycelium with aerial hyphae indicate it belongs to *Actinomycetota* (Barka et al. 2016). Chemotaxonomy analyses revealed that the strain MW-W600-9^T^ has whole-cell sugars, diaminopimelic acids, mycolic acids and lipids characteristic of the *Nocardia* genus (Conville et al. 2018; Tamura et al. 2018; Benndorf et al. 2020). Phylogenetic analyses based on sequencing of the 16S rRNA gene and further whole-genome sequencing confirmed this and indicated that the strain is a new species. Both analyses showed that the closest validly named species are *N. asteroides*,* N. neocaleodensis* and *N. rhizosphaerihabitans*. In the 16S rRNA gene-based tree, strain MW-W600-9^T^ is at the root of the clade that comprises the mentioned species as well as other *Nocardia* species. While tree based on the core proteome, which also includes type strains from the closely related species, showed that the strain is within the clade with *N. neocaleodensis*,* N. rhizosphaerihabitans* and *N. asteroides.* The closest strain to the strain MW-W600-9^T^ is *Nocardia* sp. MH4, which at the moment has no status as a type strain and does not represent a validly named species. But both dDDH (51.1%) and ANIb (92.28%) indicated that the strain MH4 and the strain MW-W600-9^T^ are also two separate species. The cut-off values for the similarity between genomes below which they belong to separate species are 70% and 95% for dDDH and ANIb, respectively (Wayne et al. 1987; Jain et al. 2018). Comparison of the strain MW-W600-9^T^ with two related type strains, *N. rhizosphaerihabitants* DSM 101726^T^ and *N. asteroides* DSM 43757^T^ by chemotaxonomic features and metabolic potential further strengthened its status as a new species. Based on the data obtained, we suggest that all 5 species shared a common ancestor, with *N. neocaleodenesis* diverging first and later separation between *N. rhizosphaerihabitans* and the rest of the species forming the clade. Speciation between the strains MW-W600-9^T^, MH4 and *N. asteroides* took place after that and could be connected with different niches which each of the species colonized. Especially, that both strains MW-W600-9^T^ and MH4 were isolated from hydrocarbon rich niches, coal mine and soil near oil pump respectively (Tanase et al. 2013). This hypothesis could be assessed in future research by performing chemotaxonomic and metabolic comparative analysis of all the members of the clade. As the strain *Nocardia* sp. MH4 is not available via culture collections, we could not perform a chemotaxonomic comparison between it and other species.

Members of *Nocardia* are mostly associated with pathogenic strains (Brown-Elliott et al. 2006; Mehta and Shamoo 2020; Traxler et al. 2022). However, they were isolated from different sources including environmental samples, microbiomes of animals and plants (Zhang et al. 2008; Tanvir et al. 2016; Thawai et al. 2017; Ghodhbane-Gtari et al. 2019; Benndorf et al. 2020; Nouioui et al. 2022). Comparison of *Nocardia* genomes showed that on average environmental, non-pathogenic strains have slightly larger genomes than more pathogenic strains (7.8 Mbp vs. 7.4 Mbp) (Nouioui et al. 2022). Additionally, most genomes from pathogenic strains are in the range 6–7 Mbp, which is the case, for example, in *N. asteroides* (Redenbach et al. 2000) and *N. farcinica* (Ishikawa et al. 2004). Isolate’s genome with size approx. 7.8 Mbp is closer to the average value for non-pathogenic strains. Furthermore, phylogenetic analyses showed that the strain is more closely related to non-pathogenic *N. rhizosphaerihabitans* (Ding et al. 2018) than to *N. asteroides*, which is an opportunistic pathogen (Brown-Elliott et al. 2006).

Analysis of the whole genome of the strain MW-W600-9^T^ detected, based on the used tool, between 6557 and 7551 CDS, which is around the average number of 6880 genes per genome found in *Nocardia*. Noticeably, a large variation in the number of genes is observed in members of the genus, ranging from 4682 to 9695 (Eripogu et al. 2025). Functional annotations of the strain MW-W600-9^T^ genes classified between 34 and 40% of detected proteins. This indicates a higher number of unassigned hypothetical proteins (66 − 60%) than 20–45% reported in other studies (Xu et al. 2021; Cai et al. 2022; Collins Fairclough et al. 2023; Eripogu et al. 2025). It also suggests a large number of genes with potentially novel functions.

*Actinomycetota* are recognized as producers of a plethora of secondary metabolites (Barka et al. 2016). Especially, genus *Streptomyces* is known for that (Belknap et al. 2020). But while in *Streptomyces* about 2–4% of the genome is dedicated to secondary metabolism, a recent study has shown that *Nocardia* have 7% of its genome related to SMs production (Otani et al. 2022; Nikolaidis et al. 2023; Eripogu et al. 2025). The genus is a vastly unexplored source of new metabolites, which can have biological activities (Eripogu et al. 2025; Nonthakaew et al. 2025). For example, Nouioui et al. (Nouioui et al. 2022) have recently shown that from 36 regions with BGCs in the *N. alni* ncl2 genome, 16 (44.44%) have similarity below 20% and 14 (38.89%) show no similarity to clusters of known compounds. An analogous situation is in the strain MW-W600-9^T^, where from 22 BGCs regions, 12 (54.55%) have similarity below 20% to known compounds, and 3 (13.64%) show no similarity. Even though BGCs in *Nocardia* are to a large extent unknown, the genus is a source of important SMs with biological activities (Shigemori et al. 1998; Igarashi et al. 2000; Kelly and Townsend 2005; Sun et al. 2009). However, only a few reports show antimicrobial activity of *Nocardia* (Li et al. 2015; Jalali et al. 2016). In the case of our isolate, no activity against either bacteria or fungi was observed in the tested conditions. It is important to remember that SMs production in *Actinomycetota* is tightly regulated, and multiple factors may influence it (Xia et al. 2020); therefore, a lack of activity in particular conditions does not exclude it completely. Future research on stimulation of SMs production, as well as other activity tests, e.g. versus cancer cells, could fully elucidate strain potential in this aspect.

Potential for degradation of aromatic compounds has been reported for several *Nocardia* strains (Hocinat et al. 2019; Yang et al. 2019; Azadi and Shojaei 2020; Shi et al. 2022). Many of them have been isolated from environments enriched in such compounds (Khomenkov et al. 2008; Yang et al. 2019; Shi et al. 2022). Our strain was isolated from underground coal mine water. As coal seams have formed from dead organic matter, they contain a multitude of complex organic matter, including aromatic compounds with different oxidation states, humic acid, etc. (Sekhohola et al. 2013). The ability to degrade such compounds is therefore crucial for microorganisms that thrive there (Sekhohola et al. 2013; Shi et al. 2022). Functional annotation of the MW-W600-9^T^ genome detected 9 genes that are important for the degradation of aromatic compounds at early stages of degradation pathways. Eight of these genes are oxygenases, either mono- or dioxygenases, crucial enzymes for cleavage of aromatic ring (Khomenkov et al. 2008). Both enzyme groups were found in *Nocardia* species with the ability to degrade aromatics. Baek et al. (Baek et al. 2006) have found gene *catA*, encoding catechol 1,2-dioxegenase in *Nocardia* sp. H17. The strain H17 was able to degrade 23.8% of the aromatic fraction in crude oil during 6 days of incubation. In strain *Nocardia* sp. Y48 gene, *pobA*, encoding *p*-hydrobenzoate 3-monooxygenase, determined the strain’s ability to grow on phenol as a single source of carbon (Yang et al. 2019). The ninth gene is haloalkane dehalogenase, which suggests the strain’s potential to metabolize haloorganic compounds (Kunka et al. 2018). The substitution of a halogen atom can take place at any step of degradation (Pimviriyakul et al. 2020), so the strain might be able to perform dehalogenation after cleavage of the aromatic ring. It cannot be excluded that other genes involved in the early stages of aromatic compounds degradation are not present in the strain MW-W600-9^T^. They might not be detected because, as mentioned above, the majority of the strain’s genes lacked functional prediction.

These rare and valuable metabolic features of the strain could be utilized by humans for the degradation of aromatic xenobiotics. Potential to remove organic compounds by the strain MW-W600-9^T^ from culture medium has been tested on BPA and BPS, and aromatic haloorganics 4CP and IOX. Bisphenols and 4CP are dangerous contaminants found in environments (Noszczyńska and Piotrowska-Seget 2018), while IOX is used as a contrast in medical imaging; it is resistant to degradation, and its accumulation in the environment makes it an emerging pollutant (Nowak et al. 2020). The strain MW-W600-9^T^ was able to remove BPA from culture medium with 43.73% reduction of BPA concentration after 21 days. The continuous decrease in BPA concentration suggests degradation, although confirmation would require identification of metabolic intermediates or mineralization studies. Bacteria able to degrade BPA have been previously isolated from environmental samples (Michałowicz 2014; de Morais Farias and Krepsky 2022; Park and Chin 2023). But to our knowledge, this is the first report of BPA degradation by *Nocardia*. Members of the genera *Bacillus* or *Pseudomonas* with the ability to degrade bisphenol A showed higher efficiency than our isolate. Strain *Bacillus* sp. GZB was able to completely degrade BPA used at the same concentration as in this study after 96 h of culture (Li et al. 2012). At the same time, strains of *Pseudomonas* sp. K-8, K-6 and KU-3 degraded 81%, 78% and 74%, respectively, over 12 days of incubation on mineral medium (Kamaraj et al. 2014). It is important to note that in the current study, we aimed for the initial evaluation of the biotechnological potential of the strain MW-W600-9^T^, while in the above examples, strains were pre-stimulated by incubation in the presence of BPA before the degradation experiment. It cannot be excluded that the isolate might show higher degradation potential or even utilization of BPA as the sole source of carbon and energy if pre-stimulated before biodegradation experiments, something that should be addressed in the future. A different situation occurred for the removal of BPS, which is more resistant to degradation than BPA (Noszczyńska and Piotrowska-Seget 2018). Slight removal of the BPS observed during the experiment indicates accumulation of the compound on biomass and later release, which could explain the lack of significant difference between the culture of the strain MW-W600-9^T^ and the abiotic control. As cells of the strain MW-W600-9^T^ are rich in mycolic acid, the hydrophobicity of their cell envelopes might increase and thus lead to increased bisphenol sorption. This hypothesis should be verified in future studies.

In the case of cultures supplemented with haloorganics, the decrease of xenobiotics in cultures of the strain MW-W600-9^T^ was comparable (27.12% and 27.38% for 4CP and 4CP + IOX cultures, respectively) and significantly different from the control culture for 4CP. For IOX, the removal was 5.1% and 9.38% for IOX and IOX+4CP cultures of the strain MW-W600-9^T^. Importantly, although relatively small, the observed decrease was not statistically significant when compared to the control for the IOX culture; however, it reached significance for the IOX+4CP culture. The concentration of 4CP dropped continuously during the experiment time, which, as for BPA, suggests degradation of the compounds, while for IOX, the situation was similar to BPS, where there was a bigger decrease in concentration of IOX after 17 days compared to 31, which suggests accumulation of the compound on the cell biomass and later release. Degradation of 4CP by *Actinomycetota* has been reported previously. Strain *Arthrobacter chlorophenolicus* A6 was able to completely mineralize of 300 mg/L of 4CP in 18.5 h (Sahoo et al. 2011). Enzymes involved in the degradation of 4CP and phenol are characterized by a high degree of similarity (Solyanikova and Golovleva 2004; Fuchs et al. 2011). Phenol degradation was reported previously for various strains of *Nocardia* (Shetty and Shetty 2016; Azadi and Shojaei 2020). Therefore, we speculate that 4CP undergoes initial aryl ring cleavage followed by dehalogenation catalyzed by haloalkane dehalogenase, although it requires confirmation. On the other hand, IOX is a chemical resistant to biodegradation due to the steric principle created by very big iodine atoms blocking access to the C-C bonds in the aromatic ring of this compound (Nowak et al. 2020; James et al. 2023). At the same time, the processes involved in the biotransformation of iodine contrast media to which IOX belongs are not well known (Nowak et al. 2020). As mentioned earlier, in culture with 4CP and IOX, the decrease in concentration of the latter was small but statistically significant in comparison to the control. This indicates co-metabolic degradation of the IOX, where 4CP acts as a substrate that induces enzymes involved in the process. Further research is required to confirm this hypothesis. They could include transcriptomics or quantitative PCR detection of the expression level of genes possibly involved with degradation, enzymatic assay for dioxygenases or dehalogenases activity measurement, as well as a detection of degradation product by means of HPLC coupled with mass spectrometry. Co-metabolism, a phenomenon where one or more easier to biodegrade compounds stimulate expression of enzymes that can catalyze the transformation of biodegradation-resistant chemicals with a similar structure, is well known (Tran et al. 2013; Xu et al. 2018; Xiang et al. 2021). Studies have also documented that 4CP, when used as a growth substrate, initiated the co-metabolic degradation of other hardly biodegradable aromatic compounds (Bo et al. 2025).The biodegradation process can be conducted by the natural or artificially created consortia of microorganisms, leading to better xenobiotic removal efficiency than the use of single strains (Díaz 2004; Cao et al. 2009, 2022; Mrozik and Piotrowska-Seget 2010; Zhang and Zhang 2022). Kumari et al. (Kumari et al. 2018) showed increased biodegradation of polyaromatic hydrocarbons by a consortium of three strains, *Microbacterium esteraromaticum* IITR47, *Pseudomonas aeruginosa* IITR48 and *Stenotrophomonas maltophila* IITR47, compared to strains acting individually. However, potential antagonistic interactions between microorganisms forming a consortium might have a negative effect on biodegradation efficiency. The lack of activity against other microorganisms by the strain MW-W600-9^T^ might make it a good candidate as a member of a microbial consortium for the degradation of xenobiotics, although it will always require proper testing, as the activity might be triggered.

### Description of *Nocardia fodinahabitans* sp. nov

*Nocardia fodinahabitans* (L. *fodina*, ‘a mine’; L. *habitans*, ‘to dwell’; N.L. participial adjective *fodinahabitans* ‘a mine dweller’ referring to the origin of the sample from which the strain was isolated, collective shaft water from a coal mine).

Gram-stain positive, aerobic, non-motile *Actinomycetota* that forms branched substrate mycelium and aerial hyphae with coccoid spores. The aerial hyphae grow orange with a white surface over time on PDA, ISP1 ISP2, AIA and AGS media, while on MSMA-C2, ISP3, ISP4 and ISP9 media, hyphae are colorless with a white surface appearing over time. The PDA, ISP1, ISP2, AIA and AGS media are stained orange-brown over time of culture; no staining was noticed for the rest of the tested media. It grows at a range from pH 5.0 to pH 10.0, temperatures 25 to 37 °C, and shows tolerance to up to 4% NaCl (w/v). The optimal growth occurs at pH 7.0, salt concentration 0.5% (w/v) and temperature 25 °C. It can utilize organic acids as a source of carbon and energy and can reduce potassium tellurite. It is resistant to rifamycin SV, lincomycin, nalidixic acids and shows tolerance to lithium chloride. Additional phenotypic properties are shown in Table 6 and on supplementary materials Fig. S4. The primary whole-cell sugars are arabinose and galactose, while the diamino acid of peptidoglycan is meso-diaminopimelic acid. Predominant fatty acids are 16:0, 18:1 ω9c and 18:0 10Me. The polar lipids are phosphatidylethanolamine, diphosphatidylglycerol, phosphatidylinositol, phosphatidylinositol mannoside and two unidentified lipids. Mycolic acids have 52–60 carbon atoms. The genome size is 7,709,572 bp and 68.79 mol% G + C content. The genome has 31 putative biosynthetic gene clusters, with several of them potentially producing new compounds. Genes involved in the degradation of aromatic compounds are present as well. The species did not show antimicrobial activity in the tested conditions, but preliminary research showed its potential to remove aromatic compounds from the culture medium.

The type strain MW-W600-9^T^ (= DSM 120649^T^ =PCM 3565^T^) was isolated from collective shaft coal-mine water sampled at 665 m bgl in Upper Silesia in Poland. The 16S rRNA gene and whole genome sequence are available in GenBank under the accession numbers: PX522239 and JBRJJC000000000, respectively.

## Supplementary Information

Below is the link to the electronic supplementary material.

Supplementary Material 1

## Data Availability

All data related to this study are presented in the manuscript. The *Nocardia fodinahabitans* MW-W600-9^T^ partial 16S rRNA gene sequence and genome sequence are available at the National Center for Biotechnology Information GenBank under accession numbers: PX522239 and JBRJJC000000000, respectively.
