# Supplementary material for: Nocardia fodinahabitans sp. nov. isolated from underground hard coal mine waters shows biotechnological potential for degradation of aromatic hydrocarbons
Source: Mol Genet Genomics. 2026 Jun 16;301(1):133. doi: 10.1007/s00438-026-02456-6 (PMC13269418; doi:10.1007/s00438-026-02456-6)
Supplement: Supplementary file 1 — Supplementary Material 1 [file 438_2026_2456_MOESM1_ESM.pdf]

Supplementary materials

Nocardia fodinahabitans sp. nov. isolated from underground hard coal mine waters shows biotechnological potential for degradation of aromatic hydrocarbons

Julia Marciniak[1], Trine Sørensen[2§], Agnieszka Nowak[1], Magdalena Noszczyńska[1], Jakub Smoliński[3], Katarzyna Machnik[3], Karolina Solska[1], Mateusz Pala[1], Frederik T. Hansen[2¶], Teis Søndergaard[2], Mariola Paściak[3*], Piotr Siupka[1**]

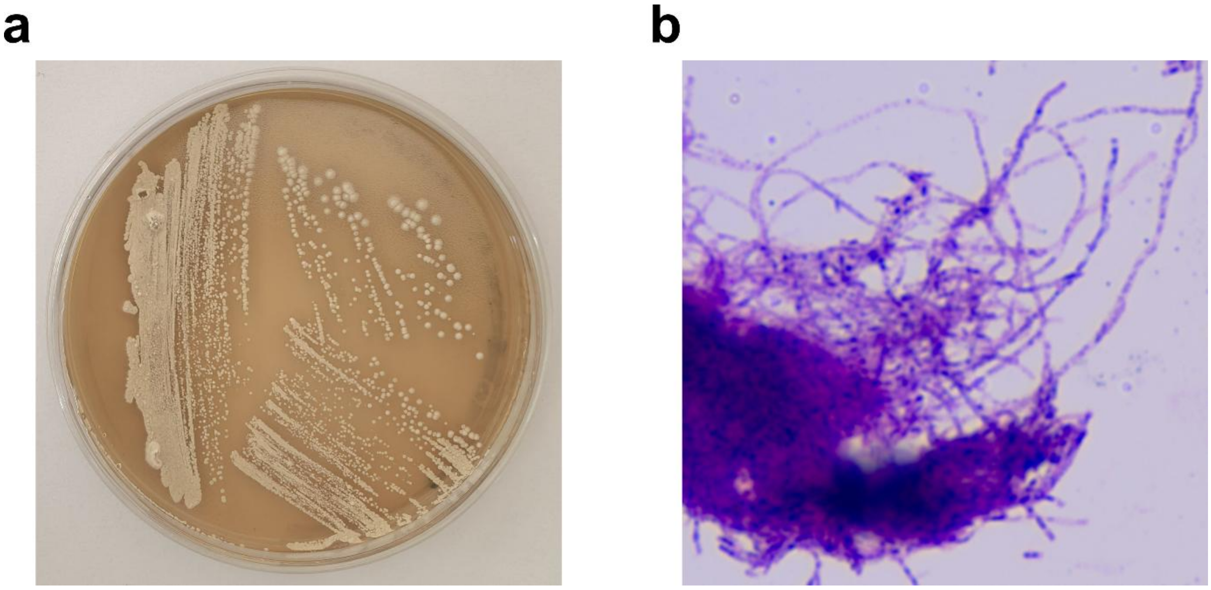

Supplementary Fig. S1 Pictures of MW-W600-9^T isolate cultured for 7 days at 25 °C in dark: (a) colonies growing on PDA plate; (b) microscopic picture of pseudomycelium after Gram-staining (magnification 1200x).

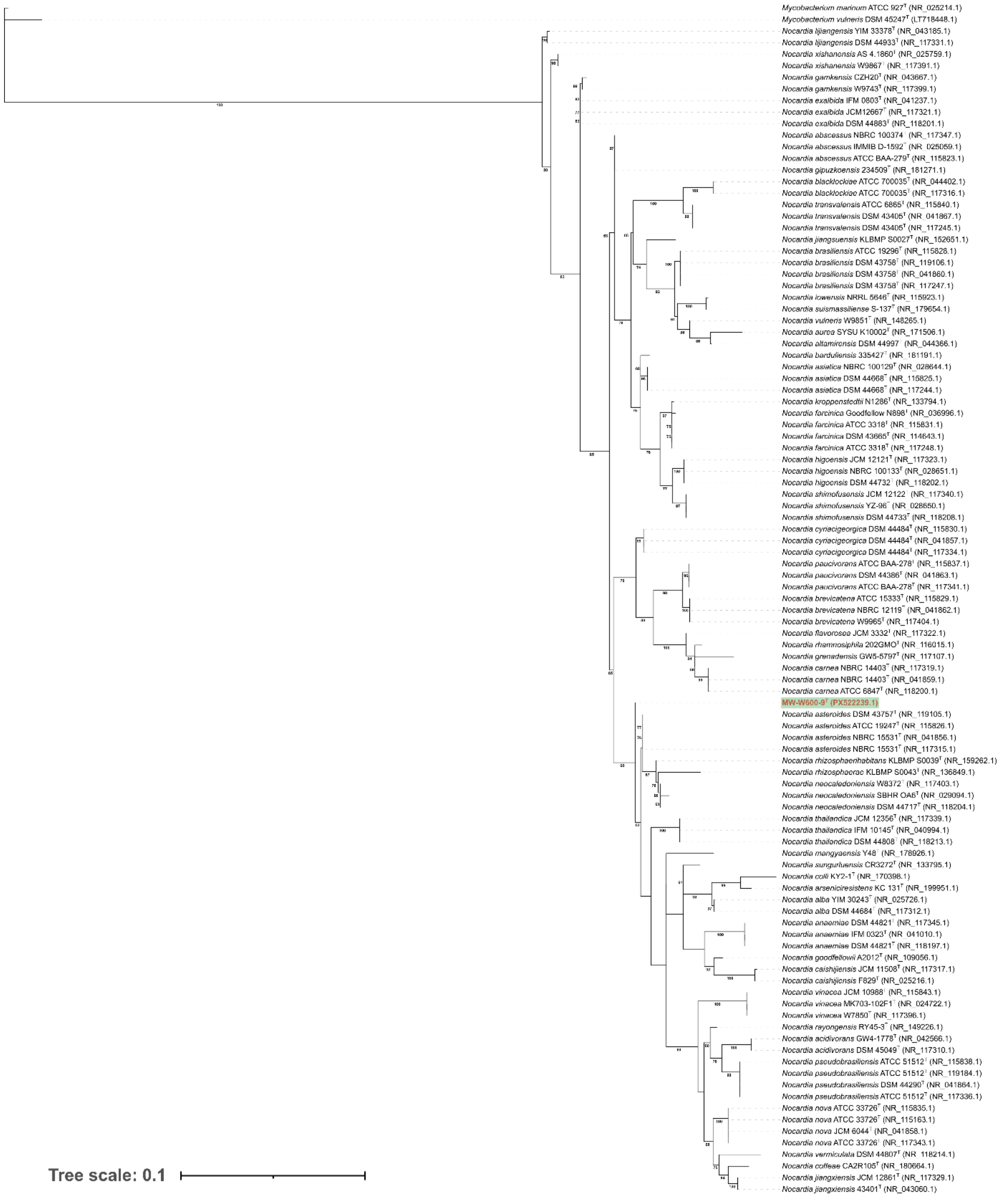

Supplementary Fig. S2 Maximum-likelihood tree of strain MW-W600-9^{T} and related type strains based on 16S rRNA gene alignment with 100 most similar sequences in NCBI's 16S/ITS database, two 16S rRNA gene sequences from *Mycobacterium* strains were used as an outgroup; numbers of branches represent bootstrap (as % of 1000 repeats), numbers in bracket are GenBank's accession numbers, node with strain MW-600-9^{T} labelled in red on green background.

Table S1. Pairwise comparison of the strain MW-W600-9^T with type-strain genomes using TYGS database.

Strain	dDDH (d0, [C.I.] %)	**dDDH (d4, [C.I.] %)**	dDDH (d6, [C.I.] %)	G+C difference, %
Nocardia rhizosphaerihabitans CGMCC 4.7329^T	50.8 [47.4 – 54.3]	**37.3 [34.9 - 39.9]**	47.5 [44.5 – 50.6]	0.37
Nocardia asteroides NCTC 11293^T	54.6 [51.1 – 58.1]	**34.9 [32.5 - 37.4]**	49.6 [46.6 – 52.7]	1.09
Nocardia asteroides DSM 43373^T	54.4 [50.9 – 57.9]	**34.8 [32.4 – 37.3]**	49.4 [46.4 – 52.5]	1.14
Nocardia asteroides NBRC 15531^T	54.4 [50.9 – 57.9]	**34.8 [32.4 – 37.4]**	49.5 [46.4 – 52.5]	1.13
Nocardia asteroides ATCC 19247^T	54.1 [50.6 – 57.6]	**34.7 [32.3 – 37.2]**	49.1 [46.1 – 52.2	1.24
Nocardia neocaledoniensis DSM 44717^T	45.4 [42.0 – 48.8]	**31.9 [29.5 – 34.4]**	41.5 [38.5 – 44.5]	0.88
Nocardia fluminea DSM 44489^T	40.5 [37.1 – 43.9]	**30.0 [27.6 – 32.5]**	37.1 [34.1 – 40.2]	1.3
Nocardia salmonicida NBRC 13393^T	40.3 [36.9 – 43.8]	**29.8 [27.4 – 32.3]**	36.9 [34.0 – 40.0]	1.76
Nocardia coubleae NBRC 108252^T	38.3 [34.9 – 41.8]	**27.8 [25.4 – 30.3]**	34.8 [31.8 – 37.8]	0.85
Nocardia coubleae DSM 44960^T	38.3 [34.9 – 41.8]	**27.8 [25.4 – 30.3]**	34.8 [31.8 – 37.9]	0.84
Nocardia thailandica NBRC 100428^T	31.4 [28.0 – 35.0]	**24.2 [21.9 – 26.7]**	28.6 [25.7 – 31.7]	2.85
Nocardia abscessus NBRC 100374^T	20.3 [17.1 – 23.9]	**22.1 [19.8 – 24.5]**	19.7 [17.0 – 22.7]	0.63
Nocardia gipuzkoensis 234509^T	20.5 [17.3 – 24.1]	**22.0 [19.8 – 24.5]**	19.8 [17.1 – 22.9]	0.3
Nocardia shimofusensis NBRC 100134^T	20.5 [17.3 – 24.1]	**21.7 [19.5 -24.2]**	19.8 [17.1 – 22.8]	0.34

C.I. – confidence interval

Table S2. Average nucleotide identity calculated using BLAST (ANIb) comparison between MW-W600-9^{T} and closely related strains.

Genome	ANIb [%]	Aligned [%]	Aligned [bp]
Nocardia rhizosphaerihabitans CGMCC 4.7329^{T}	87.66	68.23	5260028
Nocardia neocaledoniensis DSM 44717^{T}	85.18	63.99	4933696
Nocardia asteroides DSM 43373^{T}	86.79	67.21	5181832
Nocardia asteroides NBRC 15531^{T}	86.80	67.23	5183472
Nocardia asteroides NBRC 15531 ATCC 19247^{T}	86.63	65.83	5074883
Nocardia fluminea DSM 44489^{T}	84.06	63.76	4915934
Nocardia shimofusensis NBRC 100134^{T}	76.09	42.37	3266839
Nocardia abscessus NBRC 100374^{T}	76.52	46.82	3609981
Nocardia gipuzkoensis 234509^{T}	76.71	45.79	3530380
Nocardia salmonicida NBRC 13393^{T}	83.99	65.39	5041567
Nocardia coubleae DSM 44960^{T}	82.46	58.56	4514898
Nocardia coubleae NBRC 108252^{T}	82.48	58.60	4518015
Nocardia thailandica NBRC 100428^{T}	79.52	55.00	4240098
Nocardia neocaledoniensis NBRC 108232^{T}	85.24	62.65	4829676
Nocardia farcinica IFM 10152	76.86	43.20	3330280
Nocardia farcinica DSM 43257^{T}	76.72	43.46	3350726
Nocardia higoensis NBRC 100133^{T}	76.14	41.27	3181367
Nocardia alba DSM 44684^{T}	82.59	56.79	4378402
Nocardia alba NBRC 108234^{T}	82.59	56.74	4374745
Nocardia sp. Root136	83.11	60.36	4653837
Nocardia ignorata DSM 44496^{T}	82.56	59.09	4555542
Nocardia ignorata NBRC 108230^{T}	82.57	59.09	4555676
Nocardia lijiangensis NBRC 108240^{T}	77.08	47.31	3647027
Nocardia xishanensis NBRC 101358^{T}	76.97	47.87	3690541
Nocardia pneumoniae NBRC 100136^{T}	76.58	44.35	3419196
Nocardia arthritidis NBRC 100137^{T}	76.60	44.77	3451776
Mycobacterium vulneris DSM 45247	70.38	24.52	1890559
Nocardia asteroides NCTC11293	86.81	67.42	5197774
Mycobacterium sp. NAZ190054	70.75	22.11	1704409

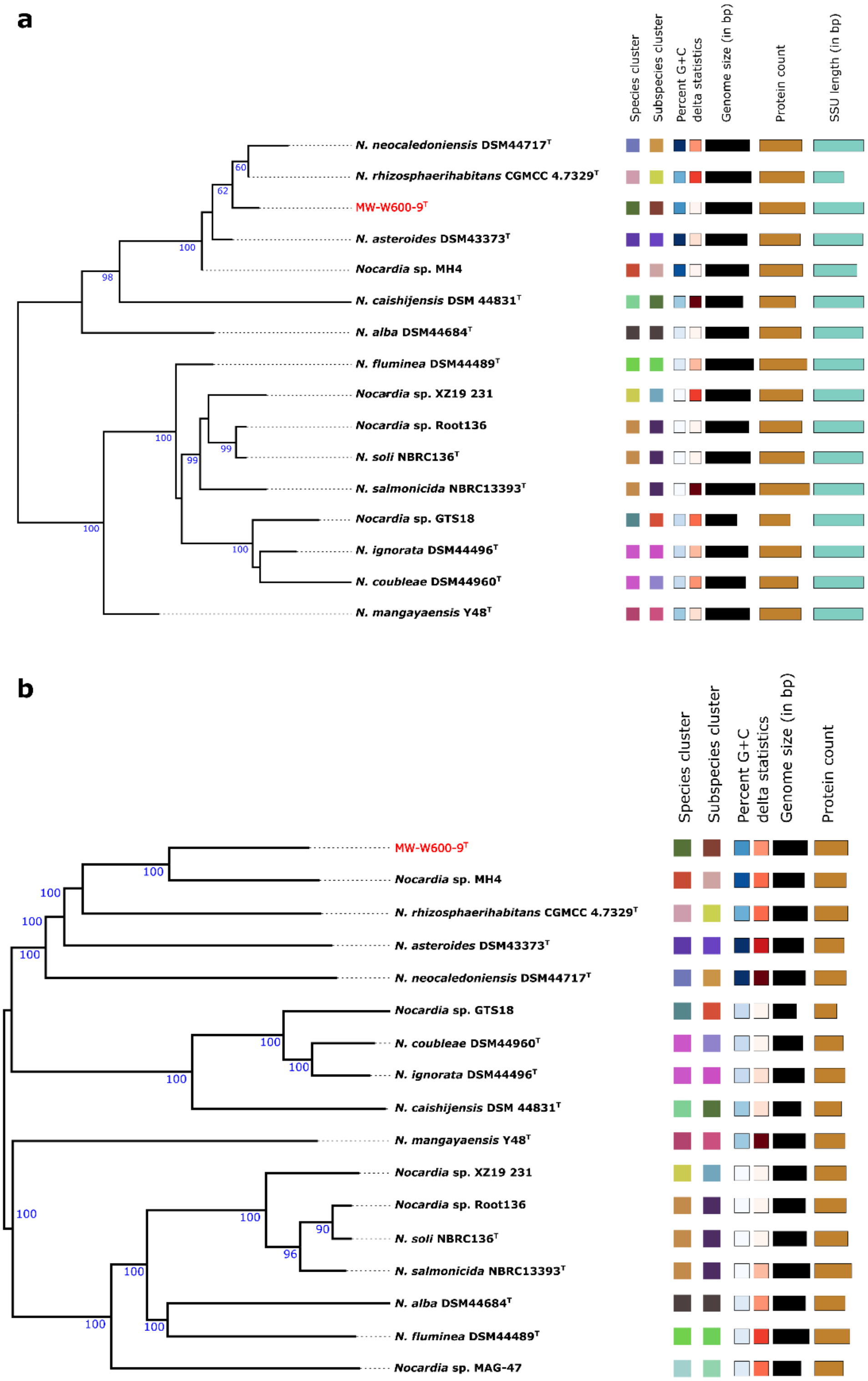

Supplementary Fig. S3 Pairwise phylogenetic trees of MW-W600-9^T and related strains; (a) based on 16S rRNA gene alignment; (b) based on whole genome sequences alignment; numbers of branches represent bootstrap (as % of 1000 repeats).

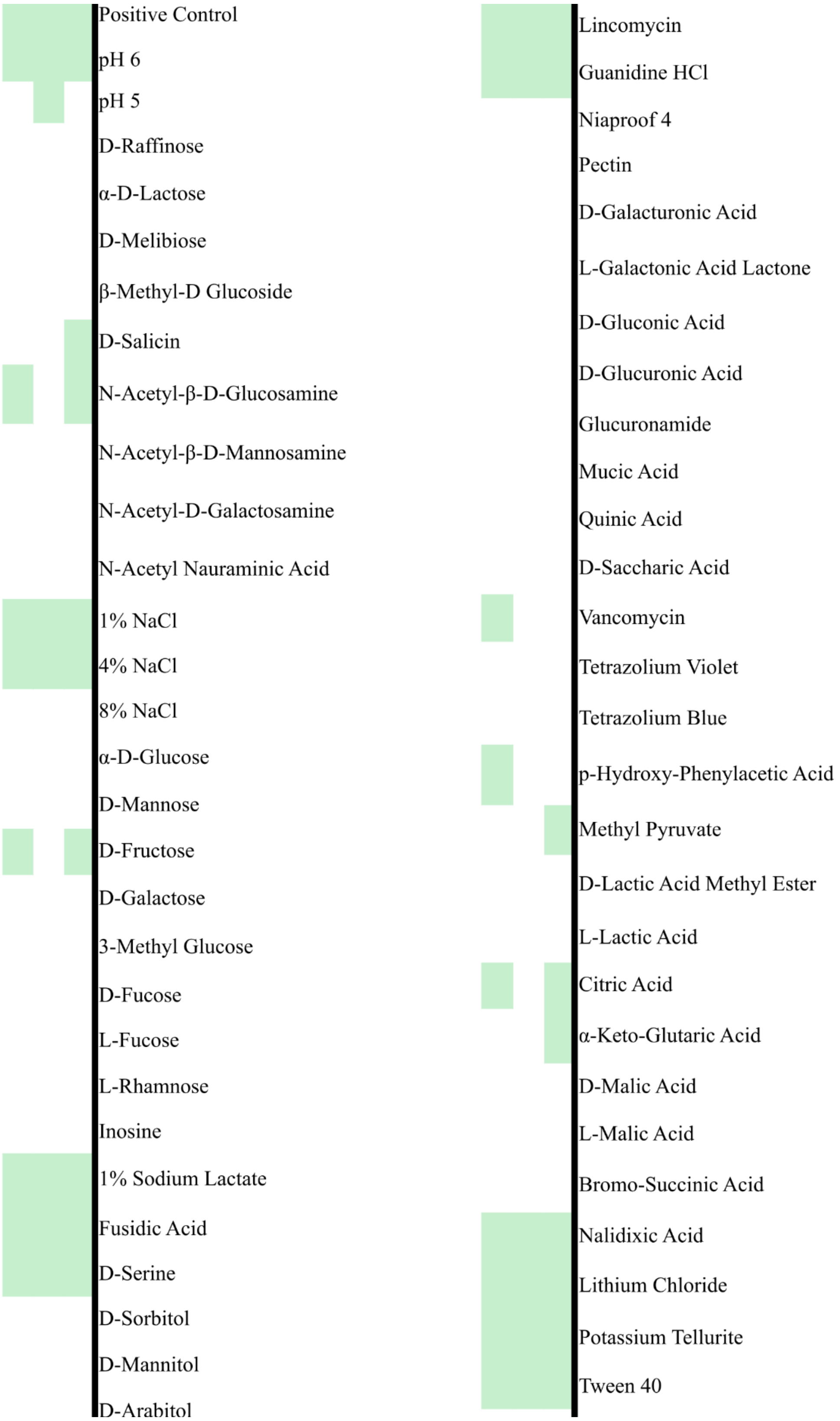

Supplementary Fig. S4 Heat-map of GEN III MicroPlate™ showing metabolic profile and growth conditions of the strains MW-W600-9^T, *N. rhizosphaerihabitans* DSM 101726^T, and *N. asteroides* DSM 43757^T; green – growth of the strain, white – no growth; for each strain, three independent repeats have been made, and the growth was classified as positive if it occurred in all repeats.

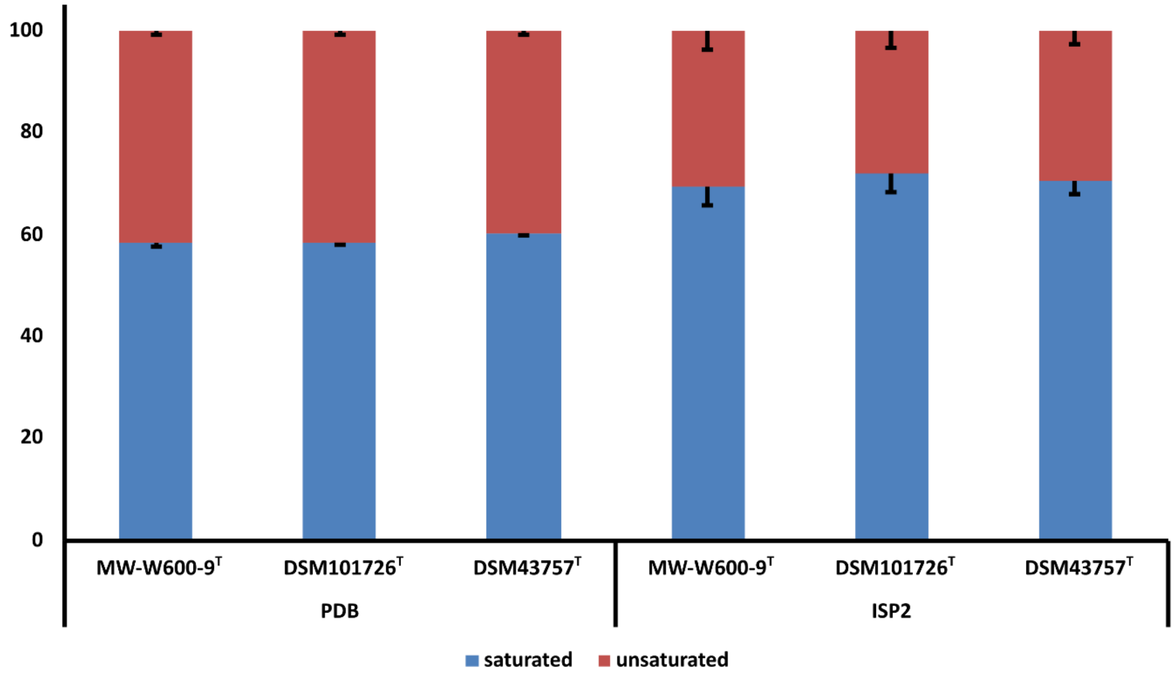

Supplementary Fig. S5 Cumulative bar plots showing the relative ratio of saturated and unsaturated fatty acids in FAME profiles of the strains MW-W600-9^{T}, *N. rhizosphaerihabitans* DSM 101726^{T}, and *N. asteroides* DSM 43757^{T}; means of three independent repeats are shown; error bars show standard deviations.

Table S3. The whole cell fatty acid profile of MW-W600-9^T, and related type strains grown on two different media. Relative amount [as %] of each acid is shown as an average of three independent repeats.

Fatty acid	PDB			ISP2		
	**MW-W600-9^T**	**DSM101726^T**	**DSM43757^T**	**MW-W600-9^T**	**DSM101726^T**	**DSM43757^T**
09:0	0.00	0.00	0.00	0.00	0.00	0.02 ± 0.04
10:0	0.00	0.00	0.00	0.00	0.00	0.02 ± 0.03
12:0	0.00	0.02 ± 0.04	0.05 ± 0.06	0.14 ± 0.12	0.00	0.23 ± 0.24
14:0	0.73 ± 0.07	1.00 ± 0.06	0.85 ± 0.34	0.66 ± 0.13	0.58 ± 0.24	0.61 ± 0.16
15:0 *iso*	0.00	0.00	0.00	0.00	0.00	0.02 ± 0.03
15:0 *anteiso*	0.00	0.00	0.00	0.00	0.00	0.04 ± 0.07
16:0	32.98 ± 0.68	32.46 ± 0.78	36.52 ± 21.28	30.34 ± 2.16	28.20 ± 4.20	32.04 ± 3.65
16:0 2OH	0.00	0.00	0.00	0.00	0.00	0.03 ± 0.06
16:0 N alcohol	0.02 ± 0.03	0.00	0.11 ± 0.02	0.11 ± 0.18	0.00	0.07 ± 0.13
17:0	1.62 ± 0.25	2.1 ± 0.28	3.37 ± 0.43	2.01 ± 0.36	1.06 ± 0.06	1.9 ± 0.35
17:0 *anteiso*	0.00	0.00	0.02 ± 0.03	0.00	0.00	0.07 ± 0.12
17:0 *iso* 3OH	0.00	0.00	0.00	0.22 ± 0.37	0.00	0.00
17:0 10Me	1.601 ± 0.23	0.43 ± 0.03	0.33 ± 0.06	1.53 ± 0.34	0.17 ± 0.01	0.08 ± 0.13
18:0	3.16 ± 0.1	12.46 ± 1.58	12.13 ± 2.85	5.06 ± 2.47	13.59 ± 2.73	10.85 ± 2.11
18:0 3OH	0.62 ± 0.07	0.35 ± 0.04	0.07 ± 0.10	0.00	0.00	0.00
18:0 10Me	17.34 ± 0.34	9.27 ± 0.36	6.14 ± 1.34	27.62 ± 2.05	26.60 ± 4.51	21.69 ± 2.18
19:0	0.03 ± 0.05	0.13 ± 0.01	0.18 ± 0.04	0.00	0.06 ± 0.00	0.09 ± 0.15
19:0 10Me	0.00	0.00	0.00	0.17 ± 0.30	0.00	0.00
19:0 *iso*	0.12 ± 0.20	0.00	0.00	0.00	0.00	0.00
20:0	0.2 ± 0.02	0.33 ± 0.04	0.6 ± 0.08	1.47 ± 0.81	1.53 ± 0.52	2.67 ± 1.22
13:1 at 12-13	0.00	0.00	0.00	0.00	0.00	0.26 ± 0.46
15:1 ω5*c*	0.01 ± 0.02	0.00	0.05 ± 0.03	0.15 ± 0.17	0.00	0.00
15:1 ω8*c*	0.2 ± 0.03	0.13 ± 0.01	0.00	0.00	0.00	0.52 ± 0.56
15:1 *iso* F	0.02 ± 0.03	0.00	0.00	0.00	0.00	0.00
15:1 *anteiso* A	0.12 ± 0.04	0.00	0.09 ± 0.07	0.00	0.00	0.00
16:1 ω9*c*	1.17 ± 0.04	1.44 ± 0.07	0.73 ± 0.24	0.00	0.08 ± 0.13	0.00
16:1 ω7*c*/ 16:1 ω6*c*	7.32 ± 0.56	3.73 ± 0.07	5.89 ± 2.24	17.39 ± 4.72	15.11 ± 4.28	16.84 ± 4.18
17:1 ω5c	0.00	0.12 ± 0.02	0.00	0.07 ± 0.13	0.74 ± 0.12	0.00
17:1 ω7*c*	0.00	0.13 ± 0.23	0.00	0.00	0.00	0.06 ± 0.11
17:1 ω8*c*	2.67 ± 0.17	1.96 ± 0.17	2.2 ± 0.10	0.67 ± 0.59	0.00	0.00
17:1 *iso* ω9*c*	0.91 ± 0.05	0.00	0.00	1.21 ± 0.10	0.14 ± 0.13	0.38 ± 0.03
18:1 ω7*c*	1.64 ± 0.04	0.75 ± 0.11	0.39 ± 0.55	0.56 ± 0.60	0.00	0.32 ± 0.55
18:1 ω9*c*	22.48 ± 1.26	28.52 ± 0.59	26.54 ± 0.29	4.14 ± 0.66	5.43 ± 0.95	4.56 ± 0.24
18:1 *iso* H	0.00	0.00	0.00	0.12 ± 0.20	0.00	0.00
19:1 ω11*c*/19:1 ω9*c*	1.29 ± 0.16	1.21 ± 0.01	0.78 ± 0.16	0.98 ± 0.16	1.11 ± 0.06	0.90 ± 0.06
19:1 *iso* I	0.24 ± 0.05	0.00	0.00	0.00	0.00	0.00
18:2 ω6,9c/18:0 *ante*	0.09 ± 0.08	0.00	0.07 ± 0.10	0.00	0.00	0.06 ± 0.11
18:3 ω6*c* (6,9,12)	0.00	0.00	0.04 ± 0.06	0.00	0.00	0.06 ± 0.10
20:1 ω7*c*	0.39 ± 0.03	0.00	0.05 ± 0.07	0.00	0.00	0.00
20:1 ω9*c*	2.68 ± 0.55	1.7 ± 0.22	0.76 ± 1.08	2.48 ± 0.79	2.41 ± 2.09	0.97 ± 1.68
20:2 ω6,9c	0.00	1.71 ± 0.25	2.06 ± 0.17	2.37 ± 0.62	2.84 ± 0.63	4.45 ± 0.43
20:4 ω6,9,12,15*c*	0.34 ± 0.14	0.04 ± 0.08	0.00	0.54 ± 0.47	0.34 ± 0.30	0.18 ± 0.32
Saturated / unsaturated	1.41	1.41	1.52	2.26	2.55	2.38

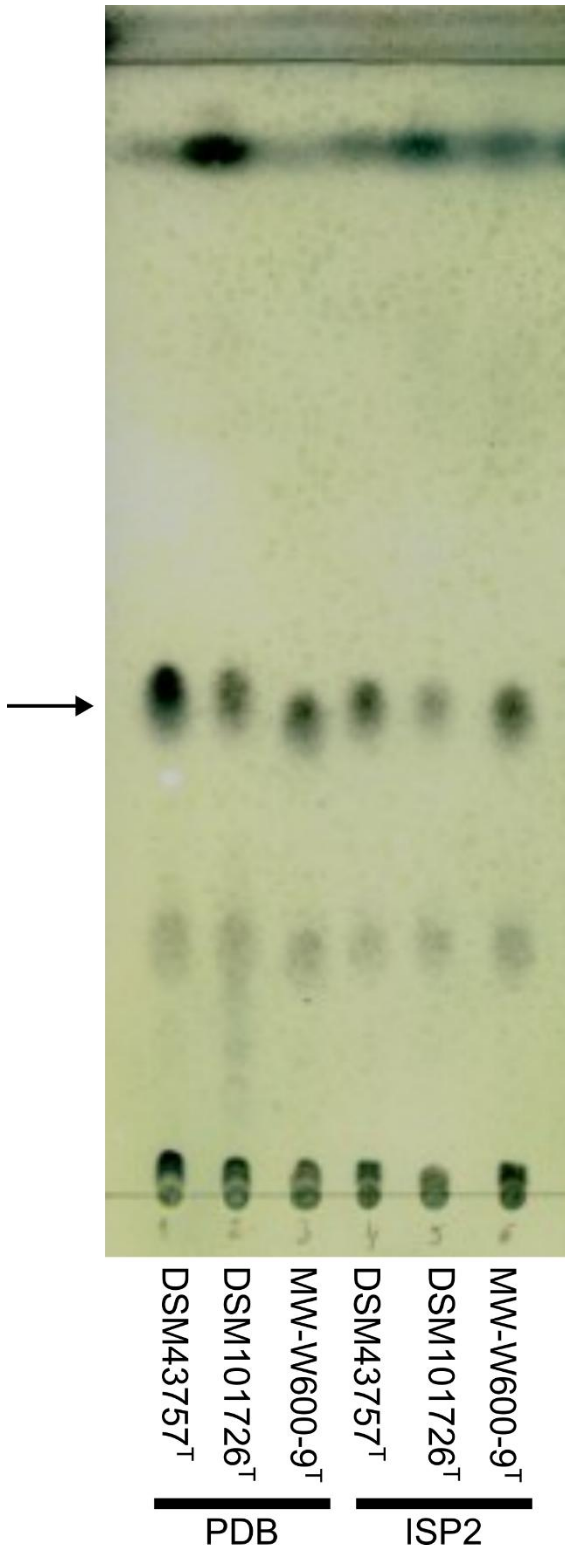

Supplementary Fig. S6 Thin layer chromatogram of mycolic acid methyl esters isolated from the MW-W600-9^{T}, *N. rhizosphaerihabitans* DSM 101726^{T}, and *N. asteroides* DSM 43757^{T} strains grown on PDB and ISP2 medium; solvent system: hexane-diethyl ether (85:15, v/v) 3 runs; detection made using phosphomolybdic acid reagent.

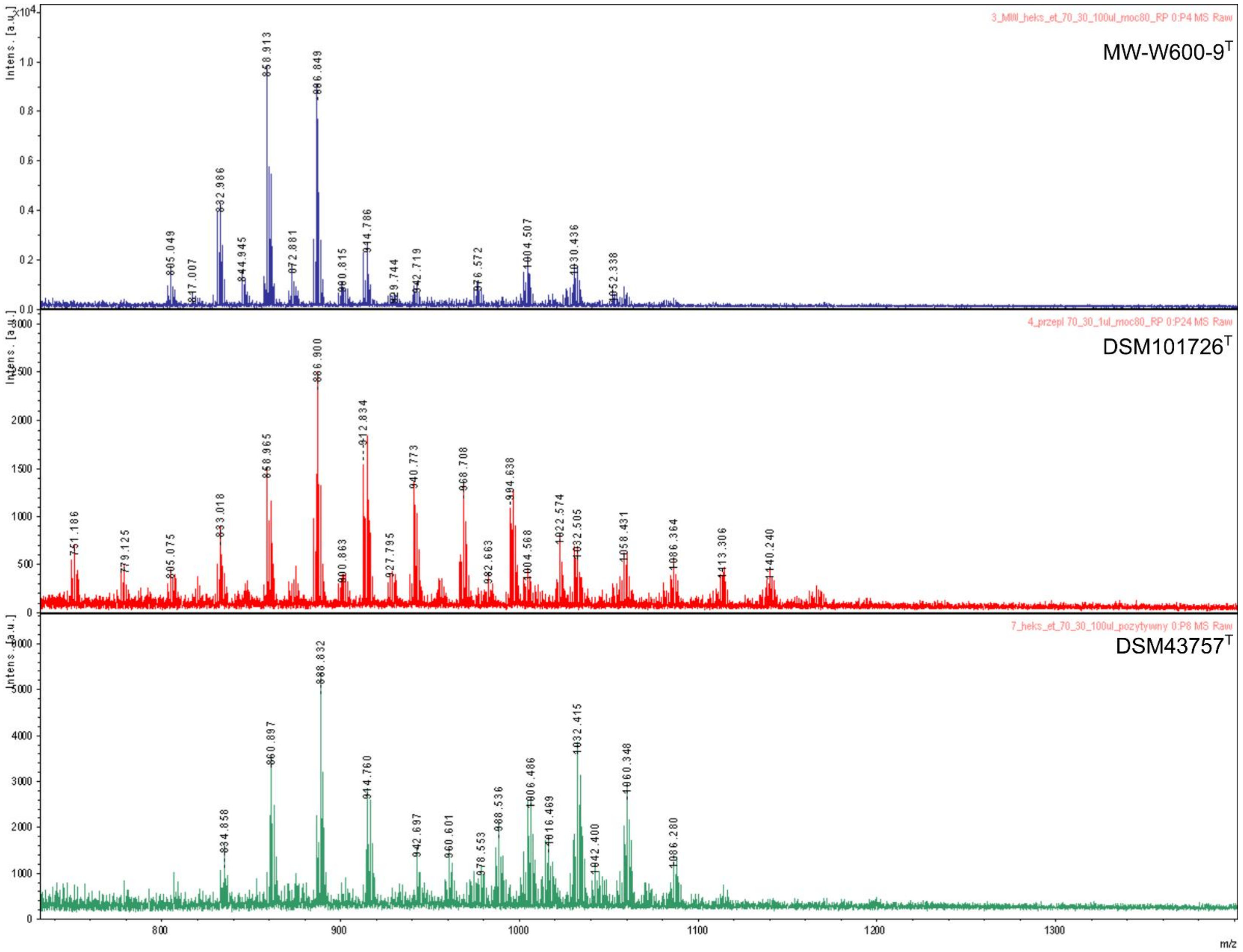

Supplementary Fig. S7 MALDI-ToF mass spectra of mycolic acid methyl ester from the strains MW-W600-9^{T}, *N. rhizosphaerihabitans* DSM 101726^{T}, and *N. asteroides* DSM 43757^{T}; matrix was norharmane in chloroform-methanol; positive ion detection mode was used.

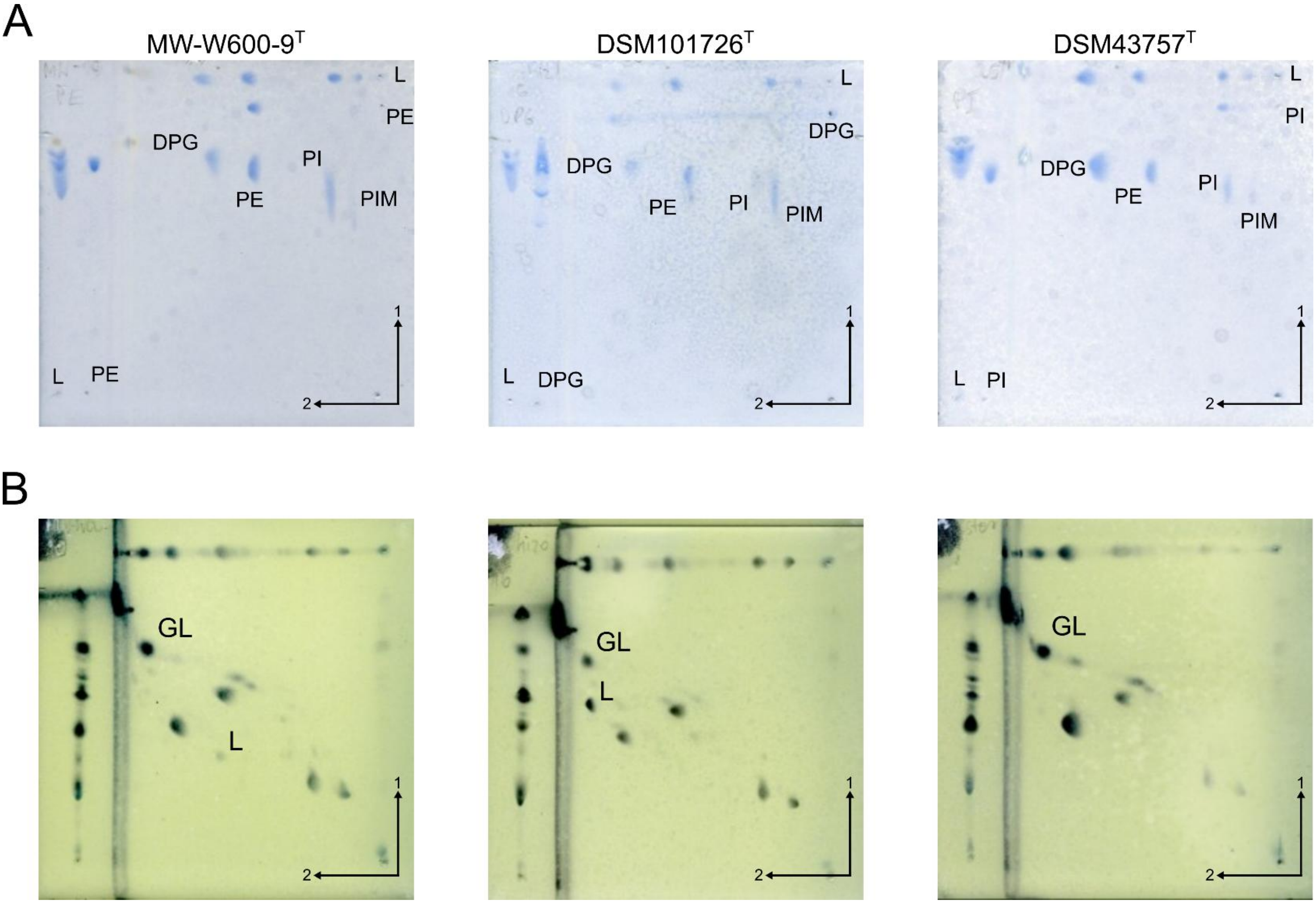

Supplementary Fig. S8 Two-dimensional thin-layer chromatograms (2D-TLC) of polar lipids from the strains MW-W600-9^{T}, *N. rhizosphaerihabitans* DSM 101726^{T}, and *N. asteroides* DSM 43757^{T} grown on ISP2 medium. Solvent systems: chloroform-methanol-water (65:25:4 v/v/v) was used for the first dimension and chloroform-methanol-acetic acid-water (80:12:15:4 v/v/v/v) was used for the second dimension; (A) phospholipids detected using Dittmer & Lester reagent; (B) total lipids detected using the phosphomolybdic acid reagent; PE - phosphatidylethanolamine; DPG - diphosphatidylglycerol; PI - phosphatidylinositol; PIM - phosphatidylinositol mannoside; GL - glycolipid; L-unknow lipid.

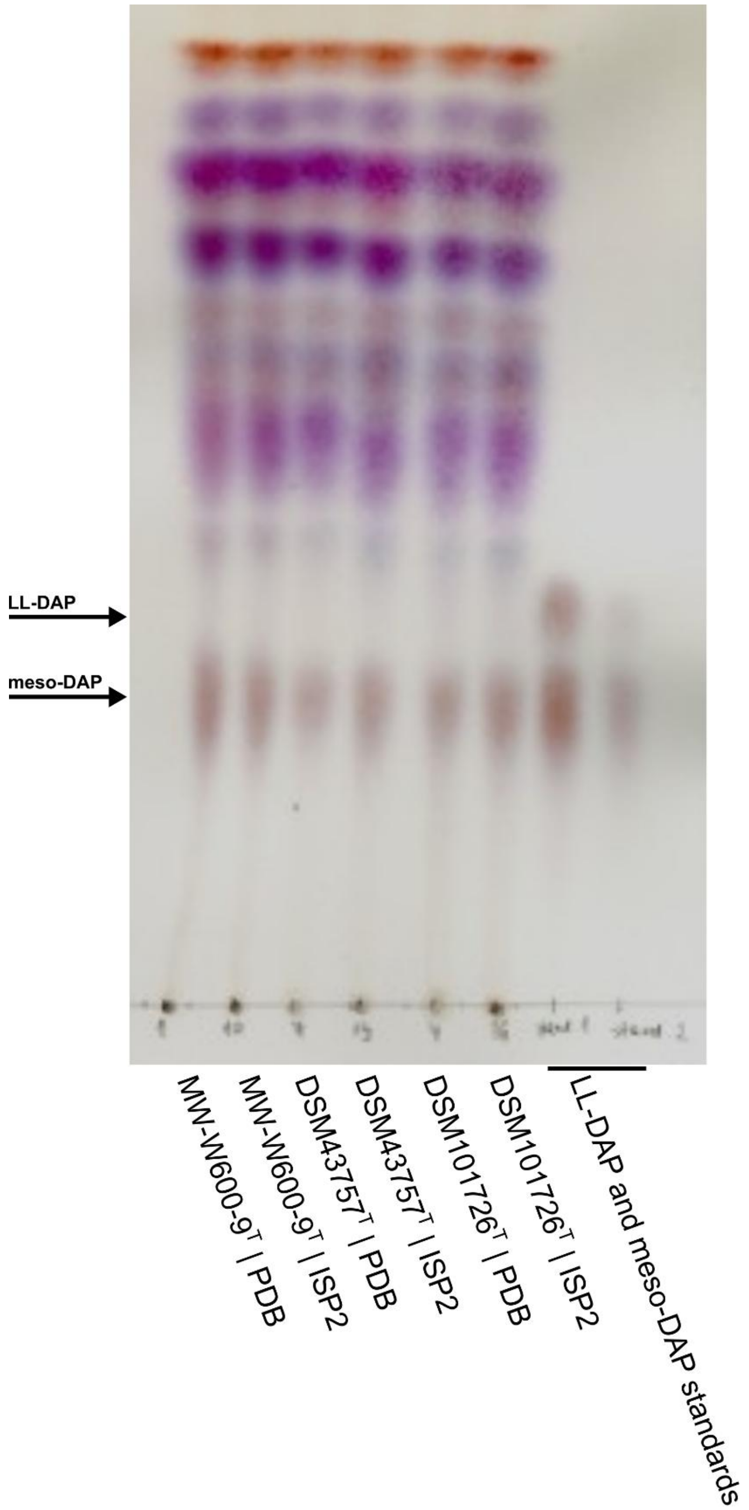

Supplementary Fig. S9 Thin layer chromatograms of whole-cell hydrolysates of the strains MW-W600-9^{T}, *N. rhizosphaerihabitans* DSM 101726^{T}, and *N. asteroides* DSM 43757^{T} growing on PDB or ISP2 medium. Solvent systems: methanol-pyridine-10M HCl-water (80:10:2.5:17.5, v/v/v/v), two runs, detection made using ninhydrine reagent; LL-DAP – L,L-diaminopimelic acid; meso-DAP – meso-diaminopimelic acid

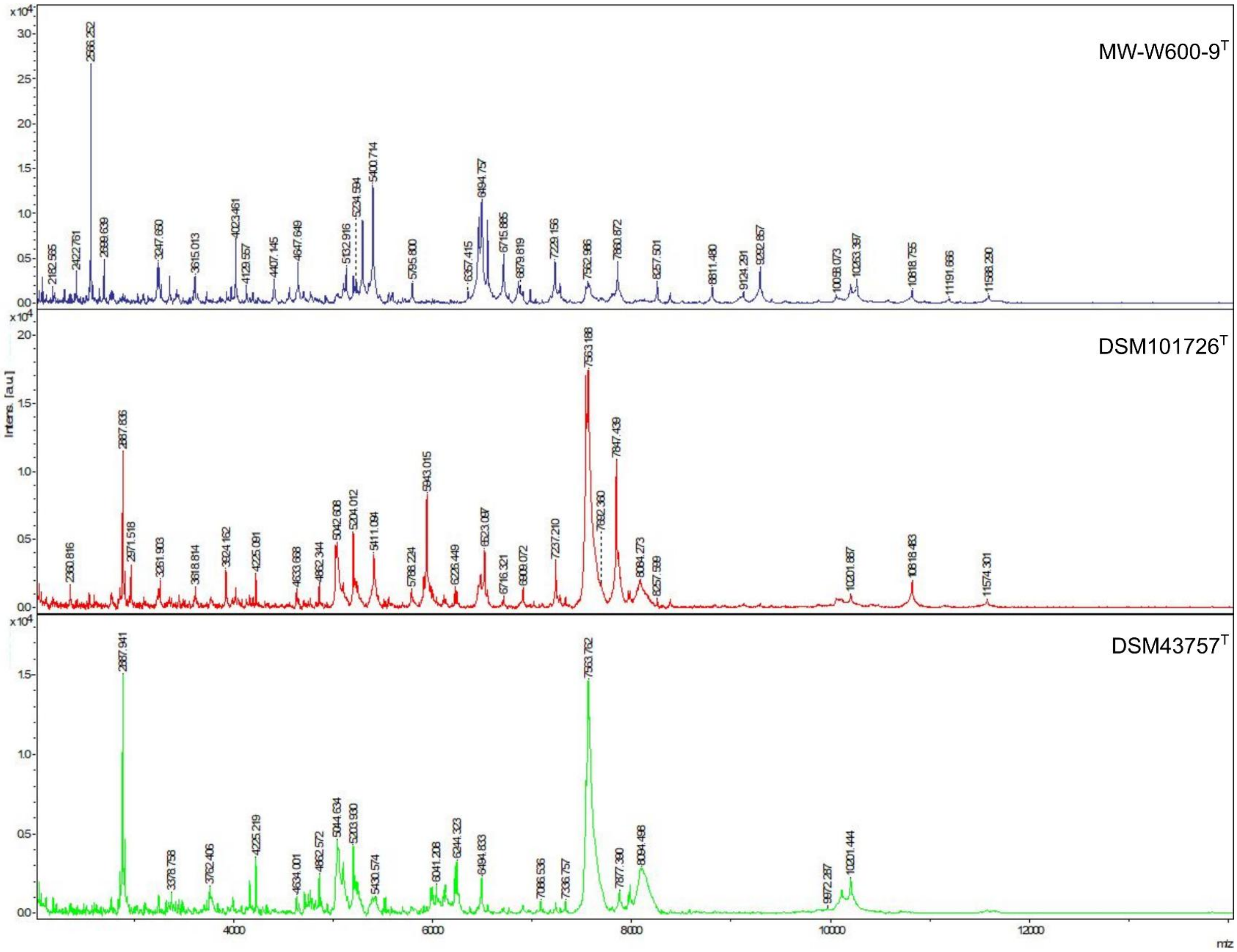

Supplementary Fig. S10 MALDI-ToF mass spectra of protein profiles of the strains MW-W600-9^T, *N. rhizosphaerihabitans* DSM 101726^T, and *N. asteroides* DSM 43757^T. All strains were cultivated on blood agar for 4 days at 25 °C.
